# Molecular Aromaticity from a Magnetic Perspective

**DOI:** 10.1021/acs.jpclett.6c02131

**Published:** 2026-08-29

**Authors:** Dage Sundholm, Qian Wang

**Affiliations:** Department of Chemistry, Faculty of Science, 3835 University of Helsinki, P.O. Box 55, A.I. Virtasen aukio 1, FIN-00014 Helsinki, Finland

## Abstract

The aromaticity concept was initially introduced to describe compounds that resemble benzene. Aromaticity was later extended to describe general ring-shaped molecules with delocalized conjugated bonds having enhanced thermodynamic stability, small bond length alternation, particular reactivity, and distinct magnetic properties. In this Perspective, we focus on various aspects of the magnetic criterion of aromaticity. Molecular aromaticity is assessed by using calculations of the magnetically induced current density (MICD) susceptibility. The degree of aromaticity is determined by calculating the strength of the magnetically induced ring current (MIRC), and the aromatic nature is obtained from the circulation direction of the MIRC vortex. The size limits of aromaticity and antiaromaticity are investigated by calculating the degree of (anti)­aromaticity of *trans*-annulene rings of increasing size. We show how magnetic shielding densities that are calculated from MICD susceptibilities can be used to understand the spatial origin of nuclear magnetic resonance (NMR) shielding tensors. We consider the topology of molecular structures and how it affects MICD. Orbital contributions to the MIRC, group theoretical aspects, and aromatic bonding of antiaromatic rings are also discussed.

## Graphical abstract

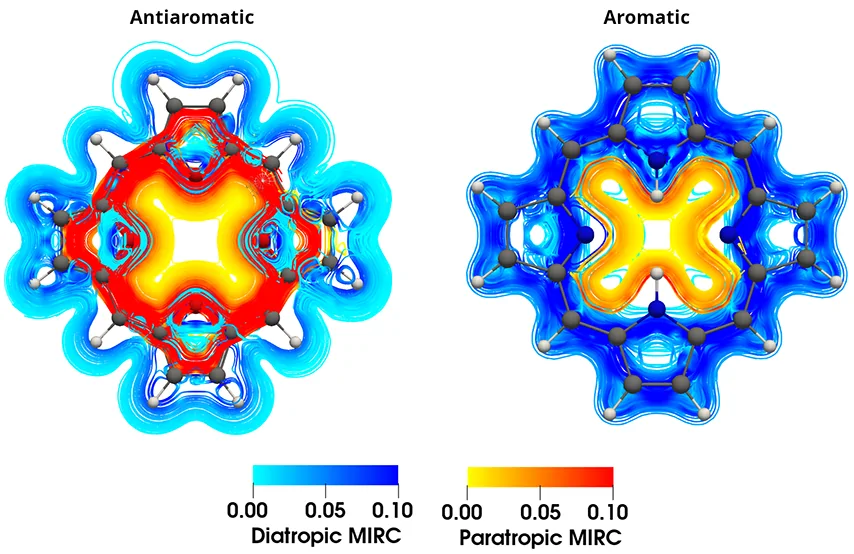

## Introduction

Aromaticity: Quo Vadis is the title of an article in *Chemical Science*, where different aspects of aromaticity were discussed by a dozen scientists active in the field.^1^ The IUPAC definition^2^ of aromaticity identifies four main means to assess the aromatic nature of molecules, namely energy, reactions, structure, and magnetic properties.^3,4^ Since these criteria do not unambiguously identify aromatic molecules and how aromatic they are, a large number of computational methods have been developed for determining the degree of aromaticity, which have unfortunately not made the aromaticity definition clearer.^5,6^ The confusion has even lead to a suggestion that aromaticity is a multidimensional property, a notion that weakens the aromaticity concept.^7,8^ Hoffmann wrote 10 years ago in a perspective article that the aromaticity hype threatens the concept but that it will survive.^9^ Among the aromaticity criteria, energy is more equal than others. However, determining various aromatic stabilization energies is complicated, both experimentally and computationally limiting its use to small molecules, whose nonaromatic reference compounds can be identified.^10,11^ Even though the energy of reference compounds is available, it is difficult to determine antiaromatic stabilization energies because antiaromatic and aromatic rings are more stable than nonaromatic ones. Otherwise, antiaromaticity would not exist. Relating reaction mechanisms and kinetic stability to the aromatic nature is, in general, complicated.^12,13^ The structural criterion, which is also assessable for large molecules and even for solid-state materials,^14^ is a reliable method to qualitatively judge whether a molecular ring of an organic molecule is aromatic or not.^15^ Bond equalization suggests that a molecule is aromatic. It is, however, a rather blunt tool after all. Antiaromatic molecules also have, in some cases, a smaller bond length alternation than nonaromatic rings due to a more effective conjugation or vice versa.^16^ The aromatic nature of inorganic clusters is difficult to assess using the structural criterion. Nuclear magnetic resonance (NMR) spectroscopy is the most useful experimental tool to determine molecular aromaticity.^17^ Since delocalized electrons in the conjugated bonds sustain a magnetically induced ring current (MIRC) in the presence of an external magnetic field oriented perpendicularly to the aromatic molecular ring,^18–21^ protons outside the MIRC are deshielded and those on the inside are shielded, that is, the inner protons are upfield shifted. MIRC effects on NMR chemical shifts of other atoms in the ring are small and difficult to determine. The MIRC and its consequences for molecular properties is the magnetic criterion of aromaticity, which we focus on in this Perspective.

## Computational MICD Studies

Computational studies of magnetically induced current density (MICD) susceptibilities are a rather new research field. To our knowledge, the first calculations were reported by Lipscomb et al. in the mid 1960s.^22^ They calculated the MICD for determining molecular magnetic properties such as nuclear magnetic shielding tensors. The main obstacle was that the calculated MICD depended on the gauge origin of the external magnetic field. The first big step forward in large-scale calculations of MICD susceptibilities was the development of a method based on a continuous set of gauge transformations (CSGT).^23^ The CSGT method was adopted and extended by the Modena/Salerno group,^24^ who called it the continuous transformation of the origin of the current density (CTOCD) method; later, it was called the ipsocentric method.^25^ Lazzeretti has reviewed the early days of MICD studies and calculations of magnetically induced ring currents (MIRC).^21^ We entered the stage in 2004, when we published the gauge including magnetically induced current (GIMIC) method,^26,27^ which has been used in a large number of applications that have been reviewed in articles and book chapters.^28–33^ The GIMIC expression for calculating MICD susceptibilities ( $J_{\alpha }^{B_{\beta }}\left(r\right)$ ) does not depend on the employed electronic structure theory level, since that information enters the density matrices in the basis-function basis. Their structure and dimension do not depend on the employed level of theory. Only the elements of the density matrices calculated at various levels of theory differ. The GIMIC expression for calculating the tensor elements of the MICD susceptibility is^26^

1 $$J_{\alpha }^{B_{\beta }}\left(r\right)=\underset{\mu \nu }{\sum }D_{\mu \nu }\left(\frac{\partial \omega_{\mu }^{*}\left(r\right)}{\partial B_{\beta }}\frac{\partial \mathrm{h̃}\left(r\right)}{\partial m_{\alpha }^{K}}\omega_{\nu }\left(r\right)+\omega_{\mu }^{*}\left(r\right)\frac{\partial \mathrm{h̃}\left(r\right)}{\partial m_{\alpha }^{K}}\frac{\partial \omega_{\nu }\left(r\right)}{\partial B_{\beta }}-\omega_{\mu }^{*}\left(r\right)\omega_{\nu }\left(r\right)\underset{\delta }{\sum }\varepsilon_{\alpha \beta \delta }\frac{\partial^{2}\mathrm{h̃}\left(r\right)}{\partial m_{\alpha }^{K}\partial B_{\delta }}\right)+\underset{\mu \nu }{\sum }\frac{\partial D_{\mu \nu }}{\partial B_{\beta }}\omega_{\mu }^{*}\left(r\right)\frac{\partial \mathrm{h̃}\left(r\right)}{\partial m_{\alpha }^{K}}\omega_{\nu }\left(r\right)$$

where ω_μ_(**r**) and ω_ν_(**r**) are magnetic field dependent basis functions (gauge including atomic orbitals, GIAOs), *D*_*μν*_ is the one-electron density matrix, $\frac{\partial D_{\mu \nu }}{\partial B_{\beta }}$ represents the one-electron magnetically perturbed density matrices, and *ε*_α_ *_βδ_* is the Levi-Civita pseudotensor with α, β, δ ∈ {*x*, *y*, *z*}.^34^ The interactions with the external magnetic field **B** and the nuclear magnetic moment **m** ^*K*^ are given by

2 $$\frac{\partial \mathrm{h̃}\left(r\right)}{\partial m^{K}}=\left(r-R_{K}\right)\times p$$

3 $$\frac{\partial^{2}\mathrm{h̃}\left(r\right)}{\partial m^{K}\partial B}=\frac{1}{2}\left[\left(r-O\right)\;\cdot \;\left(r-R_{K}\right)1-\left(r-O\right)\left(r-R_{K}\right)\right]$$

where *h̃* denotes that the common singular denominator is omitted. **p** is the momentum operator. **R**_*K*_ is the spatial position of nucleus *K*, and **O** is an arbitrary gauge origin that disappears in eq 1 when using GIAOs. The explicit dependence on the nuclear position also cancels in eq 1, making the MICD susceptibility independent of the nuclear positions and nuclear magnetic moments.

The MICD can be calculated at all levels of theory that can be employed in calculations of NMR shielding tensors using the energy derivative approach.^35,36^ We have reported MICD calculations at the most common electronic structure levels such as Hartree–Fock (HF), density functional theory (DFT), second-order Møller–Plesset perturbation theory (MP2), coupled cluster singles and doubles (CCSD) and CCSD with a perturbational treatment of the triples (CCSD­(T)), full CCSDT,^26^ and the complete-active-space self-consistent field (CASSCF) levels.^37^ The density matrices needed for the GIMIC calculations can be obtained by calculating NMR shielding tensors using, for example, Turbomole,^38,39^ Cfour,^40^ or Gaussian.^41^ MICD calculated at the DFT level is in general accurate enough for interpretations. Calculating accurate MICDs for antiaromatic molecules requires a large share of long-ranged Hartree–Fock exchange in the functional to avoid exaggeration of the antiaromatic nature. The commonly used B3LYP functional^42^ works well in most cases for aromatic molecules.

The MICD susceptibility is as fundamental for molecular magnetic properties as the electron density is for molecular electrostatic properties. Molecular magnetic properties like magnetizabilities, NMR shielding tensors, and magnetically induced magnetic fields can be calculated from the MICD.^21^

## The Degree of Aromaticity

The degree of aromaticity can be quantified by calculating the strength of the MIRC (*I*), which can be obtained for a given direction of the external magnetic field by integrating the MICD passing through a plane that cuts the molecular ring.^26^ The plane is usually perpendicular to the ring and parallel to the direction of the external magnetic field. The MIRC is integrated numerically using

4 $$I=\int_{S}\underset{\beta \in x,y,z}{\sum }\frac{B_{\beta }}{\left|B\right|}{Ĵ}^{B_{\beta }}\left(r\right)\;\cdot \;\mathrm{n̂}\;ds$$

where *B*_β_ are the components of the external magnetic field, ${Ĵ}^{B_{\beta }}\left(r\right)$ is the MICD susceptibility, and **n̂** is the normal of the plane. Since the charge conservation condition of the MICD is almost fulfilled when using GIAOs in GIMIC calculations, the integration plane can be put anywhere around the ring. However, the numerical integration is more accurate when the plane cuts the ring between atoms. An alternative way to determine MIRC strengths is to employ Ampère–Maxwell’s law, which states that the MICD flux through a surface is proportional to the induced magnetic field along the edges of the surface.^43^ The MIRC strength can be obtained by a line integration along the center of the MIRC vortex.

5 $$I=\frac{1}{\mu_{0}}\oint_{l}\frac{B_{\mathrm{induced}}}{\left|B\right|}\;dl$$

where μ_0_ is the vacuum permeability, **B** is the external magnetic field, and **B**_induced_ is the induced one. Since **B**_induced_ in the limit of an infinitely weak **B** is obtained by multiplying with the magnetic shielding tensor (**B**_induced_ = −**σB**), the magnetic field dependent term in eq 5 can be replaced by **σ** calculated in discrete points along the edge of the surface. **σ** vanishes far away from the molecule. For example, the MIRC strength of benzene when **B** is perpendicular to the ring in the *x* *y*-plane can be obtained by integrating the *zz*-component of the nucleus independent chemical shift (NICS_*zz*_)^44^ along the *C*_6_ symmetry axis. MIRC pathways can be determined in molecules with fused molecular rings by using appropriate integration planes or positions for line integrations. The MIRC strength of benzene is ca. 12 nA/T regardless of the employed level of theory. In contrast, the strength of the paratropic MIRC of the antiaromatic tetraoxa-isophlorin is −62.5 nA/T at the DFT level using the B3LYP functional, whereas at the MP2 level it is −27.4 nA/T.^45^

Calculations of the MICD pathways can provide information about the Clar structures of benzenoid polycyclic aromatic hydrocarbons (PAHs). The Clar structure is the resonance structure of the PAHs with the largest number of disconnected aromatic π-sextets,^46^ which is the energetically most stable resonance structure. Computational and experimental studies confirmed Clar’s rule for hexabenzocoronene, which has one Clar ring in the center and six Clar rings along the perimeter.^26,47–49^ Calculations of the MICD of circum­[*n*]­coronenes (*n* = 1, 2, 3, ...) showed that circumcoronene has the same Clar rings as hexabenzocoronene.^47,50^ In contrast, the ring in the center of circum[2]­coronene sustains a paratropic MIRC, but it has no Clar rings.^50^ The MIRC strengths of circumcoronene and circum[2]­coronene are shown in Figure 1. Circum[3]­coronene has seven Clar rings in the circumcoronene moiety in its center, whereas the two surrounding shells of benzenoid rings have no Clar rings.^50^ The MICD is dominated by the global MIRC along various pathways around the larger circum­[*n*]­coronenes.^50^ Pictures of the MIRC pathways and MIRC profiles calculated at the CAM-B3LYP and MP2 levels using split-valence polarization quality (def2-SVP) basis sets^51^ are shown in the Supporting Information (SI). The direction of the MIRC of the ring in the center alternates with increasing size of the circum­[*n*]­coronenes, whereas the rest of the MIRC pathways flow in the classical direction for the studied circum­[*n*]­coronenes.^50^

**Figure 1:**
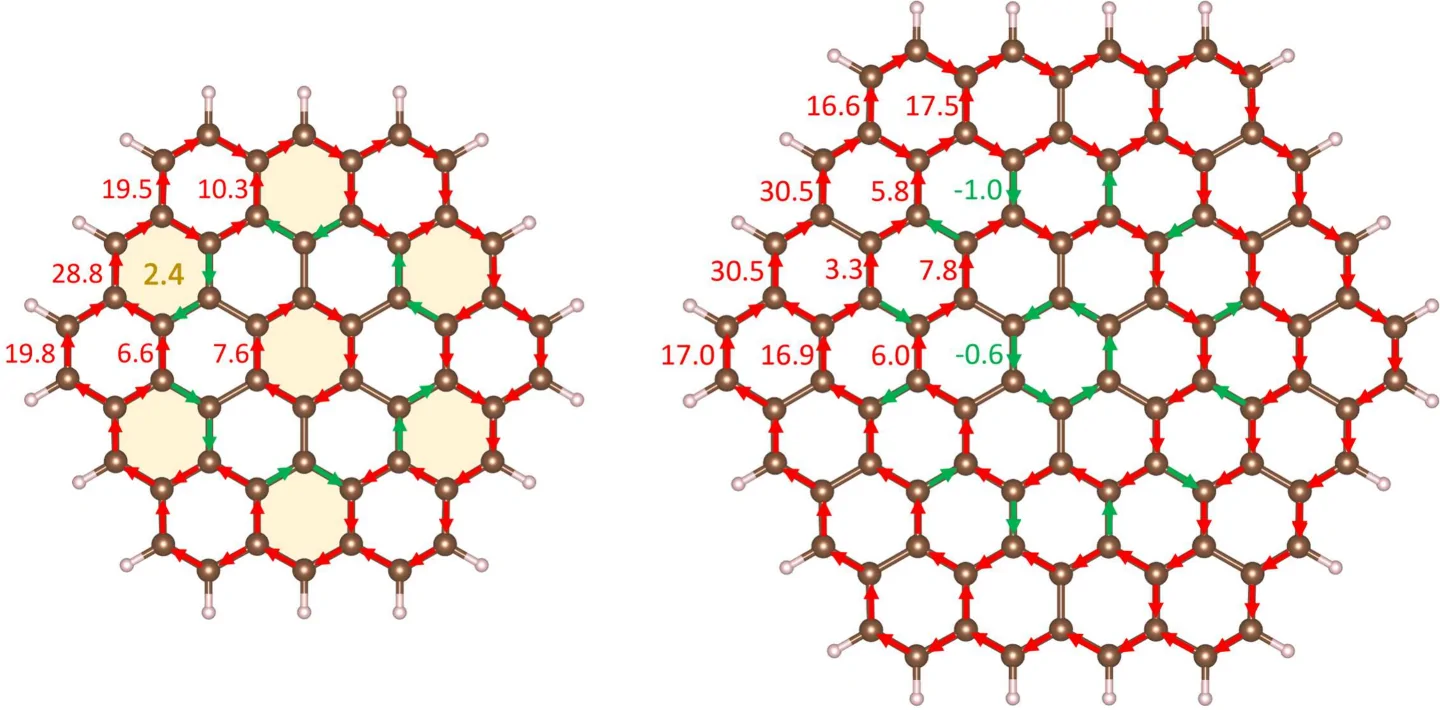
MIRC strengths (in nA/T) calculated at the CAM-B3LYP/def2-SVP level along various pathways in circumcoronene and circum[2]­coronene are reported. The local MIRC strength (in nA/T) of the yellow Clar rings is also given.

The accuracy of the numerical integration is higher than other uncertainties of the MICD calculations. The GIMIC expression fulfills the charge conservation condition in the limit of complete basis set.^26^ The divergence in the valence region is already small for organic molecules when using def2-SVP basis sets. Most of our MICD studies have been performed with triple-ζ quality basis sets (def2-TZVP), leading to an even smaller divergence in the valence orbitals sustaining the MIRC. The MIRC strengths calculated using the def2-SVP and def2-TZVP basis sets agree well. The largest uncertainty in the integrated MIRC strengths originates from nonvertical stagnation lines of the MIRC vortices. The accuracy can be improved by integrating along the stagnation line, which is computationally challenging but feasible.^52^

## Tropicity

Tropicity is the circulation direction of MICD vortices relative to the orientation of the external magnetic field. Diatropic MICD vortices circulate in the classical direction, whereas MICD vortices in the opposite direction are paratropic. Molecular rings dominated by a diatropic MIRC are aromatic when the MIRC strength exceeds 3 nA/T, which is a threshold based or our experience.^53^ It is difficult to identify aromatic or antiaromatic properties when the MIRC is weaker than ±3 nA/T because MIRC contributions to ^1^H NMR chemical shifts are then very small and obscured by other effects. Molecular rings sustaining a significant paratropic MIRC are antiaromatic. Nonaromatic rings sustain diatropic and paratropic MIRCs of the same size that cancel. Alternatively, there is no global MIRC but only a local MICD vortex at the studied bond. The tropicity of a single ring can be easily determined from the direction of the MIRC at the integration plane, whereas for complicated molecules with many fused rings, a means to determine the tropicity is to follow the vector field around the vortex and judge from the MICD streamline around the vortex whether the MICD circulation is in the diatropic or paratropic direction.^54^

For a given direction of the external magnetic field, the MICD trajectory around the whole vortex can be calculated using the Runge–Kutta algorithm.^55,56^ The calculation starts from an arbitrary point in space (*a⃗*_*n*_), where the MICD vector in that point is *v⃗*_*n*_. The next point (*a⃗*_*n*+1_) along the trajectory can be estimated from *v⃗* in the present point by using the Runge–Kutta method. The procedure is repeated until one approaches the starting point. The trajectories must form closed loops to ensure charge conservation. Due to numerical approximations, the starting point and end point slightly differ. The tropicity in a chosen grid point is determined by calculating the cross product *d⃗*(*a⃗*_*n*_) = *v⃗*(*a⃗*_*n*_) × *v⃗*(*a⃗*_*n*+1_) in every step along the trajectory, calculating the scalar product of *d⃗*(*a⃗*_*n*_) with the vector of the external magnetic field, and adding the contributions from each trajectory step around the whole vortex. Its sign yields the tropicity of the MICD in the studied grid point. The diatropic and paratropic MICD contributions can be identified and separated by repeating the procedure for all grid points. The diatropic and paratropic contributions can then be analyzed separately.

## Magnetic Shielding Density

The elements of NMR shielding tensors are generally calculated as the second derivative of the energy with respect to the direction of the external magnetic field and the magnetic dipole direction of the nuclear magnetic moment. When the MICD susceptibility $\left({Ĵ}^{B_{\tau }}\left(r\right)\right)$ is known, the elements of the NMR shielding tensors can be calculated as the integral over ${Ĵ}^{B_{\beta }}\left(r\right)$ elements multiplied by the corresponding terms of the derivative of the vector potential of the nuclear magnetic moment with respect to the size of the magnetic moment in the limit of no magnetic moment $\left(\frac{\partial A^{M^{K}}\left(r\right)}{\partial M_{\delta }^{K}}\right)$ using eq 6.^21,22,57,58^

$$\sigma_{\alpha \beta }^{K}=\int \underset{\delta \gamma }{\sum }\epsilon_{\alpha \delta \gamma }{Ĵ}_{\gamma }^{B_{\beta }}(r)\frac{\partial A^{M^{K}}\left(r\right)}{\partial M_{\delta }^{K}}{|}_{\begin{array}{l} B_{\beta }=0\\ M_{\delta }^{K}=0 \end{array}}\;d^{3}r$$ 6

The magnetic shielding density is the integrand in eq 6, which represents the spatial contribution to the elements of the NMR shielding tensor.^33,59–62^ The spatial contribution to the NMR shielding density is a scalar function that can be analyzed using similar approaches as those used in electron density studies. The elements of the NMR shielding tensor can be interpreted by visualizing the shielding (positive) and deshielding (negative) contributions. When a planar molecule is in the *x* *y*-plane and the magnetic field is along the *z*-axis, the σ_*zz*_^*K*^(**r**) contribution of nucleus *K* is

7 $$\sigma_{zz}^{K}\left(r\right)=-\frac{\mu_{0}}{4\pi }\int \left(\frac{\left(x-R_{K_{x}}\right)}{|r_{K}{|}^{3}}{Ĵ}_{y}^{B_{z}}\left(r\right)-\frac{\left(y-R_{K_{y}}\right)}{|r_{K}{|}^{3}}{Ĵ}_{x}^{B_{z}}\left(r\right)\right)\;d^{3}r$$

In eq 7, one sees that the sign of the contribution to the tensor element depends on the relative direction of the MICD susceptibility ( ${Ĵ}_{x}^{B_{z}}\left(r\right)$ and ${Ĵ}_{y}^{B_{z}}\left(r\right)$ ) and on the sign of the vector potential terms.^61,62^ Shielding domains arise when the vectors are parallel, while antiparallel orientation leads to deshielding regions. The NMR shielding densities of H and C in benzene are shown as an example in Figure 2. The aromatic MIRC contribution is seen along the outer edge of the ring. The paratropic MIRC inside the ring also contributes to the NMR shielding of H and C.

**Figure 2:**
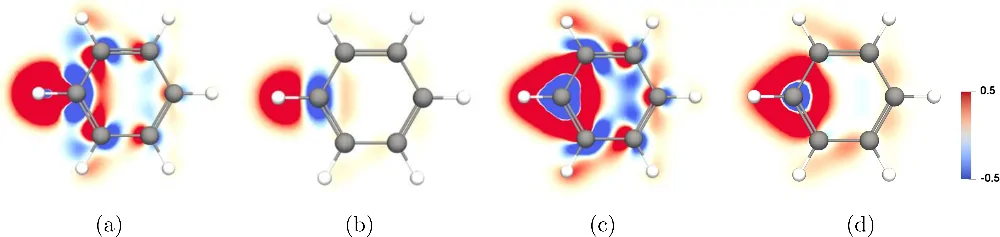
NMR shielding density for (a) H calculated in the molecular plane, (b) 1 bohr from the plane, (c) for C in the plane, and (d) 1 bohr from the plane of benzene. The calculations were performed at the B3LYP/def2-TZVP level. The (positive) shielding contributions are shown in red, and the (negative) deshielding contributions are shown in blue.

The MICD vortices are in general not centered at the studied nucleus, implying that seen from a specific nucleus, the MICD changes the relative direction and sign on the other side of the vortex origin. The contribution from an adjacent vortex changes from shielding to deshielding on the opposite sides of the center of the vortex.

The shielding density can also be used in studies of the spatial origin of NICS^44^ values and the induced magnetic field^63,64^ near molecules^60,65^ because the NICS points are virtual magnetic dipole moments probing magnetic shielding tensors and the corresponding induced magnetic field in discrete points in space.

## Size of Aromatic Rings

In an *Accounts of Chemical Research* article, Jirásek, Anderson, and Peeks rhetorically asked whether aromaticity has a size limit because they had synthesized a large macrocycle with 162 electrons in the conjugated bonds around the ring. The diameter of the ring is 5.1 nm, and even larger molecular rings may sustain an MIRC.^66^ We investigate here how large molecular rings can sustain an MIRC by calculating the MIRC strength of *trans*-annulene rings of increasing size. The rings were divided into aromatic rings with 4*n* + 2 carbon atoms and antiaromatic rings with 4*n* carbon atoms. The molecular structure of the aromatic and antiaromatic C_*n*_H_*n*_ rings belonging to the $D_{\frac{n}{2}}$ point groups were optimized at the DFT level using the B3LYP functional, the def2-TZVP basis sets, and the empirical dispersion term (D4).^42,51,67^ Calculations on the smaller members of the series show that the structures are minima on the potential energy surface. We also performed optimization of the molecular structure of the aromatic rings using the $D_{\frac{n}{2}d}$ point group resulting in equidistant C–C distances. The pseudo-π (PP) approach^68^ was used for very large rings with equidistant carbon atoms. MIRC strengths calculated for the smaller *trans*-annulene rings and for Zn-porphyrin nanorings at the PP level agree well with those obtained in all-electron calculations.^50,69^ Aromatic rings smaller than C_30_H_30_ have equidistant carbon atoms, and for them, the MIRC strength increases linearly with the number of carbon atoms. A symmetry breaking occurring at C_30_H_30_ leads to a deviation from the linear trend and a decline of the MIRC strength for larger aromatic rings. The absolute value of the MIRC strength of the aromatic C_90_H_90_ and the antiaromatic C_92_H_92_ is smaller than 3 nA/T at the B3LYP level. The C_110_H_110_ ring has a diameter of 4.3 nm and sustains an MIRC of 0.9 nA/T. The MIRC is weaker when using functionals with more HF exchange. At the CAM-B3LYP level,^70^ the absolute value of the MIRC strength of the aromatic C_50_H_50_ and antiaromatic C_48_H_48_ is smaller than 3 nA/T. We also studied the MIRC strength for rings smaller than C_30_H_30_ at the MP2/def2-TZVP level.^39,71^ The MIRC strengths calculated at the MP2 level agree well with those obtained at the DFT level using the CAM-B3LYP functional except for C_26_H_26_, whose MIRC strength at the MP2 level is about twice that calculated at the DFT levels of theory. The MIRC strength of C_50_H_50_ calculated at the MP2/def2-SVP level is 1.2 nA/T as compared to 2.8 nA/T at the CAM-B3LYP/def2-TZVP level. The MIRC strengths as a function of the ring size calculated at the MP2 level and at the DFT levels using various functionals^42,70,72^ are shown in Figure 3a and reported in the SI.

**Figure 3:**
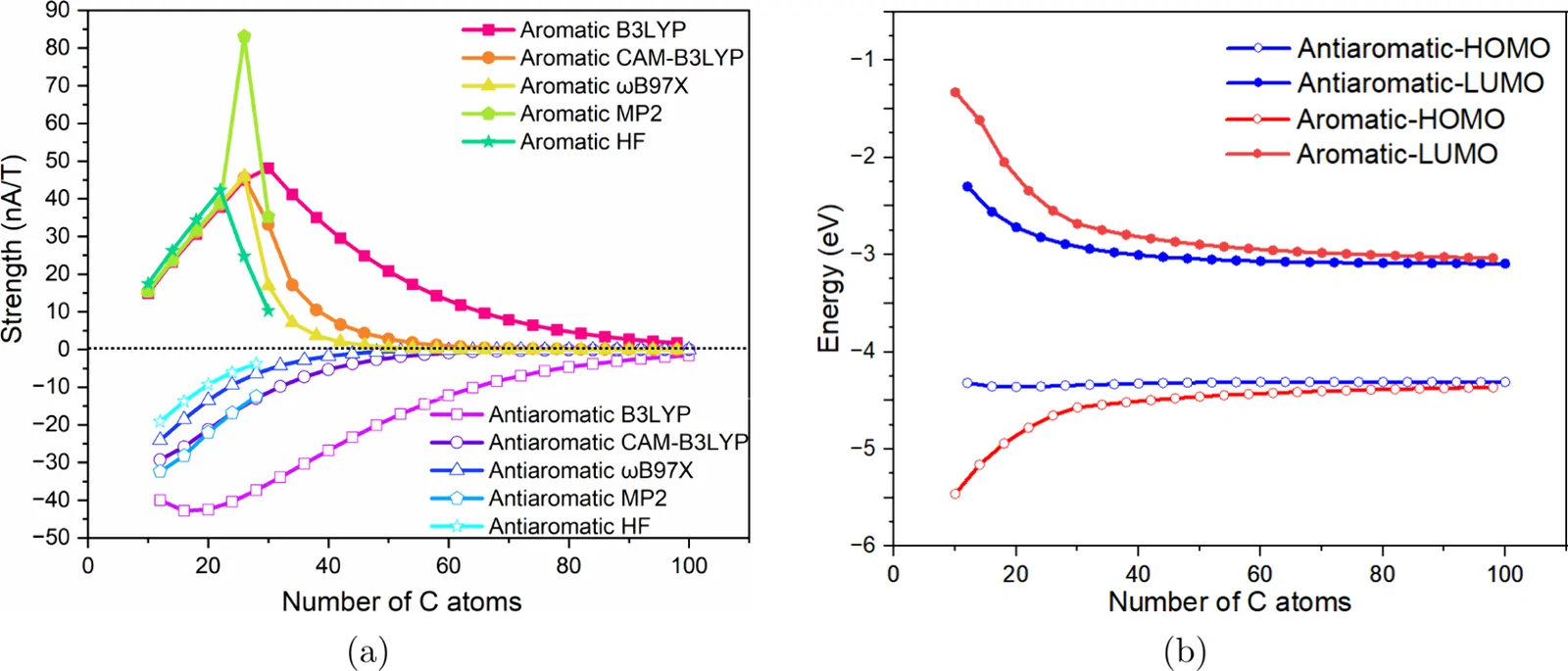
(a) The strength of the MIRC (in nA/T) of the aromatic and antiaromatic *trans*-annulene rings as a function of the number of carbon atoms in the ring calculated at various computational levels. (b) The HOMO and LUMO energies (in eV) of aromatic and antiaromatic *trans*-annulene rings calculated at the B3LYP/def2-TZVP/D4 level as a function of the number of carbon atoms in the ring.

At the B3LYP level, the MIRC strength of aromatic rings reaches a maximum value of 48 nA/T for C_30_H_30_, whereas the MIRC strength of the antiaromatic molecules is almost constant until C_24_H_24_ and then decreases with an increase in the size of the ring. At the CAM-B3LYP and MP2 levels, the aromatic C_26_H_26_ has the strongest MIRC, and for the antiaromatic rings, the MIRC strength decreases with increasing ring size. Bond length equalization plays an important role in molecular aromaticity. There is no upper limit for the size of aromatic rings with equidistant carbon atoms, as shown in the SI. Their MIRC strength increases linearly with the number of carbon atoms, which seems to continue to infinitely large rings according to the PP calculations. However, the bond length alternation appearing in the larger rings leads to a weak MIRC. Vibrational and temperature effects would affect the MIRC strengths, but they are computationally expensive for molecules of this size.

The energy gap between the highest occupied molecular orbital (HOMO) and the lowest unoccupied molecular orbital (LUMO) is 1.2 eV for large antiaromatic rings. It is almost independent of the ring size. The HOMO–LUMO gap of the aromatic rings decreases with increasing size of the ring and approaches for large rings the same value as for antiaromatic rings because very large rings are nonaromatic regardless of the number of carbon atoms in the ring. The HOMO and LUMO energies as a function of the number of carbon atoms are shown in Figure 3b.

## Topology

Aromaticity is a topological property. The aromatic and antiaromatic nature of molecular rings can often be determined by performing calculations at the Hückel level of theory. The same aromatic character is also obtained when considering that the electrons are on an infinitely thin ring or on a circular disk, which suggests that point group theoretical studies can provide information about the aromatic nature of even nonplanar molecules. A well-known example is spherical aromaticity,^73^ where electrons are thought to fill orbitals whose angular dependence is expressed using spherical harmonics. The magic numbers of the electrons for closed shells are then 2­(*n* + 1)^2^, where *n* = 0, 1, 2, ..., corresponding to 2, 8, 18, 32, 50, 72, ... electrons. It is not a coincidence that one can identify the octet rule, the 18-electron principle, and the 32-electron principle. A typical spherical aromatic molecule is C_60_^10+^ with 50 electrons in the conjugated orbitals.^74^ The MICD of C_60_^10+^ is diatropic on the inside and the outside of the molecular framework in contrast to neutral C_60_, whose MICD is diatropic on the outside and paratropic inside the molecular frame. The tetrahedral NH_4_^–^ and P_4_ belonging to the *T*_*h*_ point group have eight valence electrons fulfilling the magic number of spherical aromaticity with *N* = 1 without having a large cavity at the molecular center.^29,75^ Their MICD is completely dominated by the diatropic contribution. Tetrahedral aromaticity can be seen as a special case of spherical aromaticity. MICD calculations on the circum­[*n*]­coronene series showed that the MIRC is completely diatropic when the number of electrons fulfills the aromaticity rule for electrons on a circular disk.^50^ When the number of electrons suggests antiaromaticity, the innermost ring sustains a local paratropic MIRC, whereas the rest of the electrons fulfill the aromaticity rule, leading to a diatropic MIRC.^50^ Almost the same MICD and MIRC strengths are obtained in calculations at the CAM-B3LYP and MP2 levels of theory.

Cylindrical aromaticity can be identified for molecules that approximately belong to the *D*_∞*h*_ point group, whose parity affects the aufbau. Cylindrical aromatic molecules have an even number of occupied conjugated orbitals filled with 4*n* electrons because the orbital sequence of the aufbau reads σ_g_, σ_u_, π_u_, π_g_, δ_g_, δ_u_, ... An early example is the B_20_ double ring, which was surprisingly aromatic even though it has 20 electrons in the conjugated bonds.^29,76^

## Möbius Aromaticity

The Hückel aromaticity and antiaromaticity rules hold mainly for planar rings. Approximating the ring as a ribbon means that it is also possible to construct Möbius-twisted rings, the theory of which has been developed and reviewed.^16,77,78^ The mathematics of the topology of twisted ribbons was developed in the 1960s and is defined by the Călugăreanu–​White–Fuller theorem,^79–82^ which states that the linking number (*L*_*k*_) is the sum of the local twist (*T*_*w*_) and the global writhe (*W*_*r*_). The linking number is an integer times π, whereas π is often omitted in discussions. *T*_*w*_ and *W*_*r*_ are real positive or negative values fulfilling *L*_*k*_ = *T*_*w*_ + *W*_*r*_. *W*_*r*_ represents the deformation of the ribbon. The aromaticity rules of twisted molecular rings state that those with an even *L*_*k*_ integer have the same aromaticity rules as planar rings, whereas twisted rings with odd *L*_*k*_ integers have the opposite aromaticity and antiaromaticity rules as compared to planar rings. The *L*_*k*_ value determines qualitatively the aromatic nature, whereas the distribution between *T*_*w*_ and *W*_*r*_ affects the extent of the aromatic nature.^83^ Molecules with large *T*_*w*_ values and small *W*_*r*_ values sustain a stronger MIRC than rings with small *T*_*w*_ and large *W*_*r*_. The deformation of the ring leads to larger *W*_*r*_ values and a smaller projection area of the ring perpendicularly to the direction of the external magnetic field.

The molecular structure of the figure-eight-shaped infinitene (C_48_H_24_) has an *L*_*k*_ integer of ±2, implying that its aromatic nature should follow the Hückel rules. However, the two edges of the polyarene sustain independent MIRCs.^84,85^ The molecule can be considered to be a deformed cylindrical aromatic molecule, explaining why it sustains a net diatropic MIRC. The projection area of the outer loop is bigger than that for the inner loop. The big guy wins. Thus, the nonclassical direction of the MIRC of the inner loop cannot stop the diatropic MIRC. Figure-eight-shaped molecules with equal size of the two loops are, in general, not able to sustain a net MIRC. However, the MIRC can make shortcuts where the strands are intersecting, leading to global MIRC pathways.^54,86^

Craig introduced a new kind of aromaticity in the 1950s.^87^ He considered orbital phases in a planar molecular ring containing a transition metal. When the *d* orbitals of the transition metal contributes to the conjugation, a phase shift is introduced by the *d*_*xz*_ and *d*_*yz*_ (*dπ*) orbitals that must be corrected by another phase shift somewhere around the molecular ring. Thus, there are two phase shifts around the ring, suggesting that it has Hückel topology with an even *L*_*k*_ integer. Thus, the aromaticity rules are the same as for benzene and cyclobutadiene. The Craig-type aromaticity was forgotten until recently, when articles reporting Craig-type aromaticity were published.^88–90^ The molecular rings are planar, and calculations of topological properties of the MIRC show no Möbius twist.^91,92^ MICD calculations on the singlet ground state (*S*_0_) and the lowest triplet state (*T*_1_) of ScC_5_H_5_ show that *S*_0_ is antiaromatic and *T*_1_ is aromatic.^93^ The molecular structures are assumed to belong to the *C*_2*v*_ point group. ScC_5_H_5_ sustains a strong paratropic MICD around the Sc atom in both states, which significantly affects the NICS values near the center of the ring. The MIRC does not cross the molecular plane, as one can see in Figure 4. Thus, there is no reason to introduce any Möbius twist because the molecular ring is planar and the MICD is not twisted. Determining the topology based on the phase of the atomic orbitals can be questioned because the orbitals are hybridized when forming chemical bonds. The phase of the atomic orbitals is not even an observable, whereas areas with different phases of the molecular orbitals can be identified in scanning tunneling microscopy (STM) experiments due to the interaction with π orbitals of the tip.^94^ If the Hückel aromaticity rules cannot be employed, one has to blame something else. Calculating the number of occupied conjugated orbitals or the number of electrons in conjugated orbitals around the ring is challenging when there are transition metals in the ring. It would not be a bad idea to let Craig-type aromaticity rest in peace.

**Figure 4:**
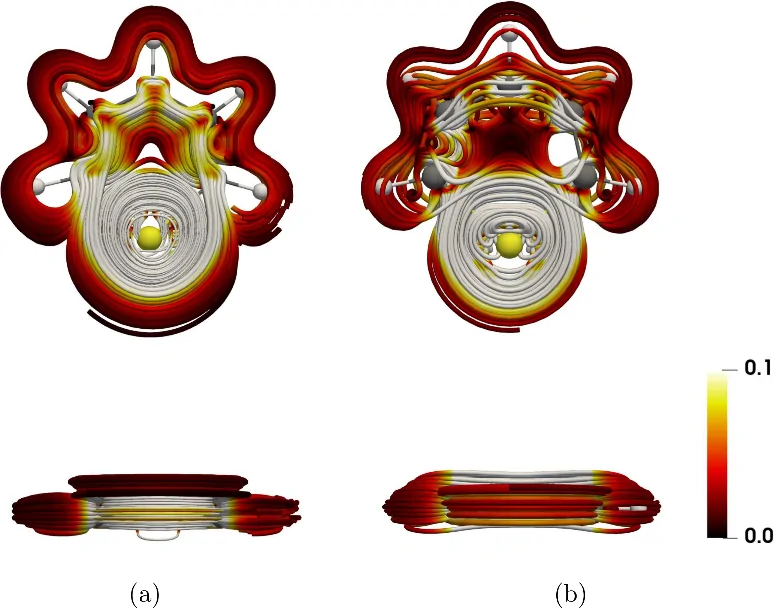
(a) The MIRC of the *S*_0_ state of ScC_5_H_5_ and (b) of the *T*_1_ state of ScC_5_H_5_. The structures are calculated at the B3LYP/def2-TZVP/D4 level. The MIRC profiles are shown in the SI.

Cumulene segments consisting of an odd number of carbon atoms have perpendicular end groups leading to helical frontier orbitals,^95–98^ whereas cumulene segments with an even number of carbon atoms have coplanar end groups and Hückel-type π orbital topology.^96^ The recently synthesized ring-shaped C_13_Cl_2_ molecule has Cl atoms attached to the *sp* ^2^-hybridized C_1_ and C_7_ atoms.^99^ The 11 *sp*-hybridized carbon atoms are divided into two segments, one with six C-C bonds and the other with seven C-C bonds. Connecting the two segments to form a ring leads formally to a 90° (π/2 rad) rotation of the *p* orbitals around the ring.^96,99^ Thus, four laps are needed to reach the starting point, which is twice the number of laps as compared to single Möbius-twisted molecules. Therefore, it was called half-Möbius topology, which is a concept that is not supported by the Călugăreanu–​White–Fuller theorem. Topology studies showed that the *L*_*k*_ integer of the helical HOMO is ±6. The *L*_*k*_ integer of the strongest streamlines of the paratropic MIRC is also ±6, where the sign depends on the chirality of the molecular structure of the C_13_Cl_2_ ring belonging to the *C*_2_ point group.^100^ The electronic structure of the antiaromatic C_13_Cl_2_ ring can be understood without introducing the half-Möbius concept.

## Aromaticity Rules

It is well-known that molecules with 4*n* + 2 electrons in the conjugated orbitals of the molecular ring are aromatic and that those with 4*n* electrons in the conjugated orbitals are antiaromatic. These aromaticity rules hold for closed-shell singlet states. The opposite rules hold for triplet states that are obtained by exciting an electron from the HOMO to the LUMO. The rules for the singlet and triplet states can be combined when realizing that one should focus not on electrons but on orbitals. The combined rule states that molecular rings with an odd number of occupied conjugated orbitals in the ring are aromatic, while antiaromatic molecules have an even number of occupied conjugated orbitals in the ring. The orbital rule has the advantage that it is simpler than the electron counting rule and can also be employed on high-spin states.^101^ Singly Möbius-twisted molecular rings have the opposite aromaticity rule, namely, molecules with an even number of occupied conjugated orbitals in the molecular ring are aromatic, and those with an odd number of occupied conjugated molecular orbitals around the ring are antiaromatic. Then, it does not matter which spin state it has. The generalized aromaticity rules can be extended to Möbius-twisted molecular rings.^102^ Then, rings with an even *L*_*k*_ integer have the same aromaticity rule as planar rings, whereas the ones with an odd *L*_*k*_ integer have the same aromaticity rule as the singly twisted ones.

## Orbital Contributions

Symmetry-based selection rules can be used for assessing which occupied orbitals contribute to the MICD susceptibility.^103^ Thus, the aromatic character can be qualitatively identified by using group theoretical arguments. When the HOMO–LUMO transition is electric-dipole (translationally) allowed, molecular rings may sustain a diatropic MIRC, that is, molecules can be aromatic when the product of the irreducible representations of the HOMO, the LUMO, and the translation operator contains the total symmetric irreducible representation. For antiaromatic molecules, the irreducible representation of the rotational operator multiplied by the irreducible representations of the HOMO and LUMO contains the total symmetric irreducible representation, that is, the transition is then magnetically dipole (rotationally) allowed, eventually leading to a paratropic MIRC.^31,104,105^ The symmetry analysis is a qualitative assessment of aromatic properties, whereas the MIRC strengths provide an extent of the aromatic nature.

Orbital contributions to the MICD susceptibility can be calculated using the CTOCD approach^21,23,25,106,107^ or by using GIMIC.^108^ Atomic and molecular orbital contributions to NMR shielding tensors can then be obtained by numerical integration.^61,62^ The orbital contributions to the MICD are not divergence free implying, that they depend in general on the orbital representation. Contributions from all orbitals in a given irreducible representation are independent of the orbital representation. However, the external magnetic field belongs to the *C*_*h*_ point group, implying that the molecular point group must be a subgroup of *C*_*h*_ and the point group of the molecular structure.^108–111^ The average contribution from one or several orbitals can be obtained as the average MIRC strength calculated for different directions of the integration plane around the molecular ring.^108^

## Strain Current

The aromaticity definition could be that all aromatic molecules sustain a net diatropic MIRC and all antiaromatic molecules sustain a net paratropic MIRC when the molecular ring is exposed to an external magnetic field perpendicular to the ring. However, molecular properties of strained ring-shaped molecules differ from those of ordinary molecular rings, leading to delocalization of the electrons in the σ orbitals,^112^ which is often recognized as σ aromaticity. 1,6-Methano­cyclo­deca­pent­aene sustains a strong MIRC due to the ring strain. Experimentally, it is not considered to be an aromatic compound because it exhibits olefinic reactivity.^113^ The average core + σ contribution to the MIRC strength is 18.4 nA/T at the DFT level using the lh20t functional^114^ and the pcSeg-2 basis sets,^115^ whereas the π contribution is only 5.6 nA/T, showing that the strain is mainly responsible for the MIRC. The strain affects the energy levels of the σ orbitals because the bonding angles are not optimal, whereas the π orbitals outside the molecular skeleton are softer and their energy levels are less sensitive to the ring strain. The MIRC in the π orbitals can be assigned to aromaticity, whereas the MIRC in the σ orbitals are due to the strain. The underlying reason for the MIRC can be judged by dividing the MIRC into σ and π contributions even though it is not foolproof.

Calculations of the orbital contributions to the MIRC of the cyclopropenium cation (C_3_H_3_^+^) and cyclobutadiene (C_4_H_4_) showed that the diatropic MIRC of C_3_H_3_^+^ is sustained in the σ and π orbitals.^108^ The paratropic MIRC of C_4_H_4_ is also sustained in the σ and π orbitals.^108^ The MIRC in the σ orbitals is probably due to the ring strain, whereas the MIRC in the π orbitals can be assigned to the aromatic nature. The HOMO – 1 (*b*_2u_) → LUMO + 1 (*b*_3u_) and HOMO (*b*_2g_) → LUMO (*b*_3g_) transitions of C_4_H_4_ are rotationally allowed, leading to a paratropic MIRC in the π and σ orbitals, respectively.

The valence σ contribution to the MIRC strength of C_3_H_3_^+^ is 5.4 nA/T, which is larger than the π contribution of 3.9 nA/T. The total MIRC strength of 10.4 nA/T is almost as large as for benzene. All orbitals except the HOMO sustain a diatropic MIRC, whose contribution is unexpectedly paratropic with an MIRC strength of −3.5 nA/T because the HOMO (e′) to LUMO + 1 (e′) transition is rotationally allowed. MIRC studies on cyclopropane (C_3_H_6_) yielded a strong MIRC in the σ orbitals and a small π contribution.^108,116,117^ Even though the MIRC strengths of C_3_H_3_^+^ (10.4 nA/T) and C_3_H_6_ (9.9 nA/T) are almost equal, the core + σ contribution to the MIRC strength of C_3_H_6_ of 9.4 nA/T is more than an order of magnitude larger than the π contribution of 0.5 nA/T. Molecules containing transition metals can also sustain MIRC in σ and π orbitals. For example, the σ contribution to the MIRC of the Co_2_O_2_ four-membered ring is 16.8 nA/T due to the ring strain, whereas the π orbitals sustain an MIRC of 7.9 nA/T.^118^

## Change in Aromatic Nature due to Face-to-Face Stacking

Antiaromatic molecular rings can form stacked aromatic dimers with a short bond distance of about 3 Å.^14,37,119–122^ The change in the aromatic nature of the stacked antiaromatic molecular rings can be understood from the irreducible representations of the frontier orbitals, which change when the molecular rings interact and formally a double bond is formed between them. The HOMO–LUMO transition (*b*_2g_ → *b*_3g_) of antiaromatic tetraoxa-isophlorin belonging to the *D*_2*h*_ point group is dipole forbidden but rotationally allowed, which leads to a strong paratropic contribution to the MIRC. The HOMO of tetraoxa-isophlorin is formally half filled. The binding combination of the two HOMOs forms the doubly degenerate closed-shell HOMO, and the antibonding combination is the LUMO of the tetraoxa-isophlorin dimer belonging to the *D*_4*h*_ point group. The HOMO–LUMO transition (*e*_u_ → *e*_g_ or *b*_2u_ → *b*_2g_ and *b*_3u_ → *b*_3g_ in the *D*_2*h*_ point group) is dipole allowed along the *C*_4_ axis (in the *z*-direction) but rotationally forbidden, suggesting a diatropic contribution to the MIRC. Since HOMO – 1 belongs to the *A*_2g_ irreducible representation of the *D*_4*h*_ point group, the HOMO – 1 → LUMO transition is dipole allowed in the *x*- and *y*-directions. The dimer sustains a diatropic MIRC when the external magnetic field is along the *x*-, *y*-, or *z*-axis. The dimer fulfills the 4*n* cylindrical aromaticity condition thanks to the reflection plane between the monomers. The smaller bond length alternation of the dimer rings also suggests a change from antiaromaticity to aromaticity when the two antiaromatic tetraoxa-isophlorin molecules form the dimer.

Aromatic binding can also be applied to other antiaromatic rings of lower symmetry. A delocalized double bond is formed between the rings because four electrons are involved in the chemical bond. Double-decker compounds with aromatic bonding can be constructed from monomers consisting of several independent antiaromatic rings, which increases the bonding strength of the stacked dimer.^14^ The aromatic bonding principle can also be extended to trimers. We performed calculations on unsubstituted norcorrole rings forming a stacked trimer. The three HOMO orbitals of the norcorroles form one binding orbital (HOMO – 4), one antibonding orbital (HOMO), and one empty orbital of the trimer. The three LUMO orbitals form one binding orbital (HOMO – 1) and two empty orbitals (LUMO and LUMO + 2), implying that four electrons contribute to the chemical bonds between the three Ni norcorroles. The frontier orbitals are reordered in the trimer, leading to an aromatic norcorrole in the middle of the trimer. The norcorrole in the middle sustains a diatropic MIRC of 21.3 nA/T and is surrounded by two antiaromatic norcorrole rings, which each sustains a paratropic MIRC of −20.3 nA/T. The trimer also sustains a diatropic MIRC of 12.6 nA/T when the magnetic field is perpendicular to the stacked norcorrole molecules, as one can see in Figure 5.

**Figure 5:**
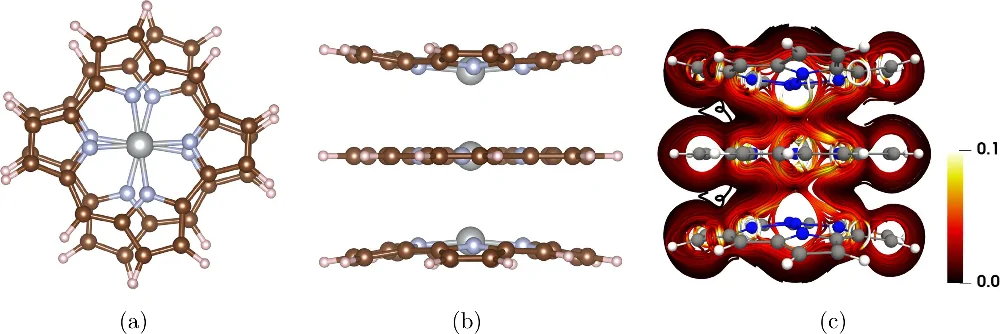
(a) The top view and (b) the side view of the molecular structure of the stacked norcorrole trimer with slightly rotated monomers. (c) The MICD calculated with the external magnetic field perpendicularly to the stack. The structure was optimized in the *C*_*s*_ point group with fixed positions of the Ni atoms taken from the X-ray structure.^123^ The dissociation energy of the trimer into monomers is 76.8 kcal/mol at the B3LYP/def2-TZVP/D4 level of theory. The dissociation energy into a dimer and a monomer is 37.3 kcal/mol at the same level of theory. The energies are corrected for basis set superposition errors. The Ni–Ni distances are 2.89 Å.

## Discussion and Conclusions

In this Perspective, we have discussed magnetic aspects of the aromaticity concept. One can argue whether the other aromaticity criteria are needed because they often introduce more confusion than information. The energetic criterion is probably the best criterion. However, calculations of aromatic stabilization energies require energies of reference compounds, which can be difficult to determine for general molecules. In addition, how can one distinguish between aromatic stabilization energy and the energy gained when nonaromatic molecules become antiaromatic without using other criteria? The structural criterion works well for a qualitative determination of the degree of aromaticity of organic molecules, whereas it is a less useful tool when assessing the aromatic nature of three-dimensional structures, as well as of molecules and clusters containing transition and heavy metals. The use of chemical reactions to assess the aromatic nature is limited. Electronic absorption spectra can provide information about the aromatic nature of porphyrinoids, whereas for general molecules, electron spectroscopy is a less useful aromaticity gauge. A large number of computational methods to determine the aromatic nature and extent have been developed. However, they still need reference systems and lead to a conflicting aromatic nature. The conflicts are resolved by introducing the unnecessary multidimensionality of aromaticity. The main advantage of the magnetic criterion is that it is strongly supported by NMR spectroscopy, which is the main experimental method to assess molecular aromaticity.

The aromatic nature of molecules can be detected by calculating MIRC strengths and pathways as well as by visualizing the MICD. The MIRC strength depends quadratically on the deviation of the direction of the magnetic field from the perpendicular orientation, implying that the exact direction is not crucial for qualitatively assessing the aromatic character.^124^ The MIRC strengths and aromatic nature of molecular rings can be rapidly obtained from the magnetic shielding tensor along the center of the MIRC vortex by line integration of Ampère–Maxwell’s law, whereas the GIMIC program can be used in extensive MICD studies including the determination of aromatic pathways. We use ParaView to visualize the MICD.^125^ For complicated molecules, the MICD can be separated into diatropic and paratropic contributions, which can be investigated separately. Calculation of orbital contributions to the MICD and the MIRC strengths shows which orbitals sustain the MIRC. The GIMIC program is open source, which including a manual can be freely downloaded.^27^ MICDs can be calculated with GIMIC at DFT levels of theory as well as at all ab initio correlated levels of theory that can be used in NMR shielding calculations employing GIAOs. The information about the magnetic response is transferred from the electronic structure code to GIMIC via density matrices. Scalar relativistic effects can be considered using effective core potentials or in all-electron scalar relativistic calculations. Spin–orbit contributions to the MICD cannot be obtained with GIMIC yet, whereas MIRC strengths can be obtained at fully relativistic levels using, for example, the mpshift^126^ program in Turbomole and Ampère–Maxwell’s law. The MICD can be calculated at fully relativistic levels with other program packages such as Dirac and ReSpect.^127,128^ The orbital response contribution to the MICD of open-shell molecules can be calculated.^129^ Open-shell MICD calculations are often more demanding than calculations on molecules with closed shells. Accurate MICD calculations on open-shell molecules require large basis set and high theory levels. NMR shielding tensors can be calculated at the complete-active-space self-consistent-field (CASSCF) level with Cfour,^40^ which is interfaced to GIMIC.^37^ CASSCF calculations are computationally expensive, and the accuracy of the MICD is limited by the size of the active space, even though very large active spaces can be used in the Cfour implementation.^130^ A large amount of HF exchange is needed when studying large antiaromatic molecules. Sometimes the MIRC strength of large aromatic molecules can also be overestimated when using, for example, the B3LYP functional with 20% HF exchange. MICD studies should therefore be performed with a few DFT functionals to assess how sensitive the MIRC strength is with respect to the amount of HF exchange in the functional. Today’s functionals with 100% HF exchange at long electron–electron distances seem to underestimate the global electron delocalization and the MIRC strengths. Calculations at DFT levels of theory are often accurate enough for interpreting the MICD. NMR chemical shielding constants and the MICD calculated at the MP2 level can be used as references when the MP2 calculations are feasible and reliable. NMR shielding tensors for organic molecules are in general accurate at the MP2/def2-TZVP level.^36^ MICD calculations on molecules containing transition metals and heavy elements are more demanding. Spin–orbit contributions to the MICD is important when aiming at accurate studies of MIRC pathways of molecules containing heavy elements and for interpreting their NMR chemical shifts.^131^ Spin–orbit contributions to the MICD caused by heavy atoms significantly affect the NMR shielding tensor of heavy atoms in the vicinity of a heavy atom (HAHA effect)^132^ and the NMR shielding tensor of light atoms near the heavy ones (HALA effect),^133^ implying that NICS values near heavy atoms calculated without considering spin–orbit effects are not reliable. Transition metals and heavy elements can also sustain a strong MIRC around the atom, affecting calculated shielding tensors in NICS points near the metal atom. MICD calculations including spin–orbit effects on large molecules representing topological insulators^134^ could provide useful information about them from a molecular perspective. Calculating MICD susceptibilities opens the avenue to computational studies of a large number magnetic properties using the numerical integration scheme implemented in GIMIC because the MICD susceptibility is the fundamental molecular magnetic response property.

## Supplementary Material



## Data Availability

The data supporting the new results reported in this article are available in the Supporting Information.

## Notes

Profiles of the magnetically induced ring current (MIRC) and MIRC strengths of circum­[*n*]­coronenes, naphthalene, and 1,6-methano­cyclo­deca­pent­aene as well as pictures of the molecular orbitals of naphthalene and 1,6-methano­cyclo­deca­pent­aene; the profile of the magnetically induced current density (MICD) and the MIRC of the Möbius-twisted C_9_H_9_^+^ ring; the diatropic and paratropic contributions to the MICD and MIRC of the lowest singlet and triplet states of the ScC_5_H_5_ ring; pictures of the molecular orbitals of ScC_5_H_5_; energies, bond lengths, and MIRC strengths of the C_*n*_H_*n*_ rings as a function of the size of the ring; and the Cartesian coordinates of the optimized molecular structure of the Ni-norcorrole trimer (PDF)
