# Supplementary material for: Molecular Aromaticity from a Magnetic Perspective
Source: J Phys Chem Lett. 2026 Aug 29;17(36):10312–23. doi: 10.1021/acs.jpclett.6c02131 (PMC13573616; doi:10.1021/acs.jpclett.6c02131)
Supplement: Supplementary file 1 [file jz6c02131_si_001.pdf]

Electronic Supplementary Information

Molecular Aromaticity from a Magnetic Perspective

Dage Sundholm* and Qian Wang*

Department of Chemistry, Faculty of Science, University of Helsinki, P.O. Box 55 (A.I. Virtanens plats 1), FIN-00014 Helsinki, Finland

E-mail: dage.sundholm@helsinki.fi; qian.x.wang@helsinki.fi

Circum[n]coronenes

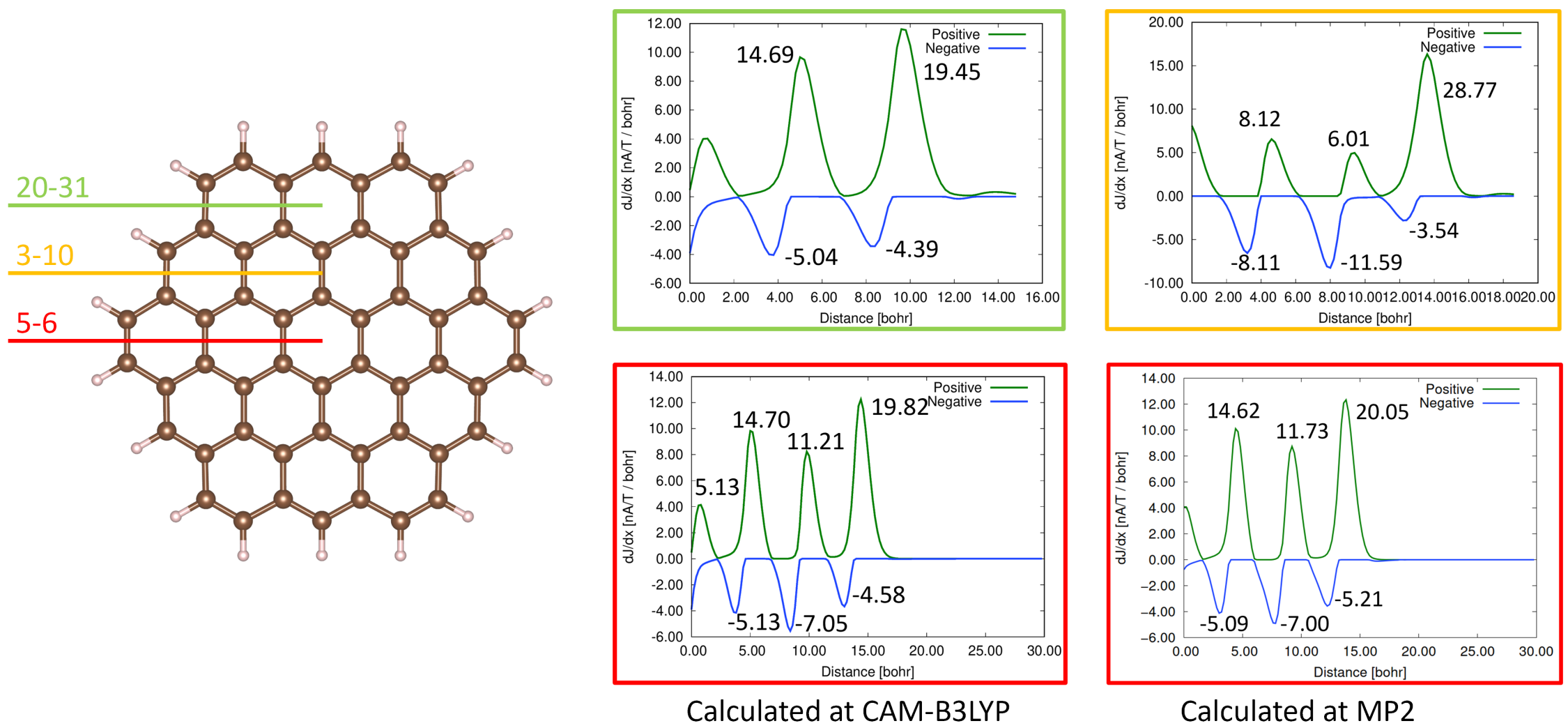

Figure S1: Magnetically induced ring-current (MIRC) profiles and integrated MIRC strengths (in nA/T) of circumcoronene calculated at the CAM-B3LYP and MP2 levels. The MIRC passing through the red plane is also calculated at the MP2 level.

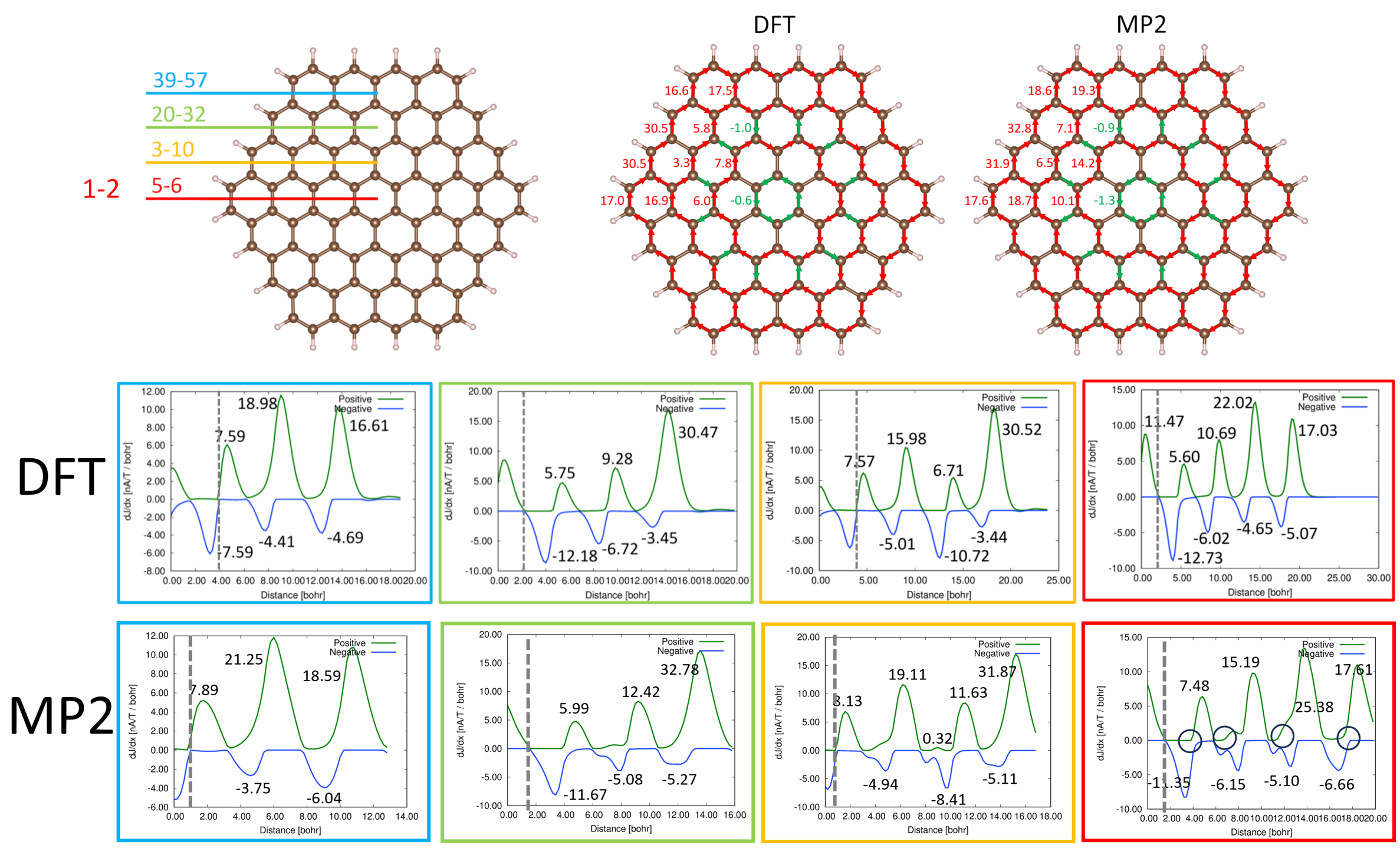

Figure S2: Magnetically induced ring-current (MIRC) profiles and integrated MIRC strengths (in nA/T) of circum[2]coronene calculated at the CAM-B3LYP and MP2 levels.

Naphthalene

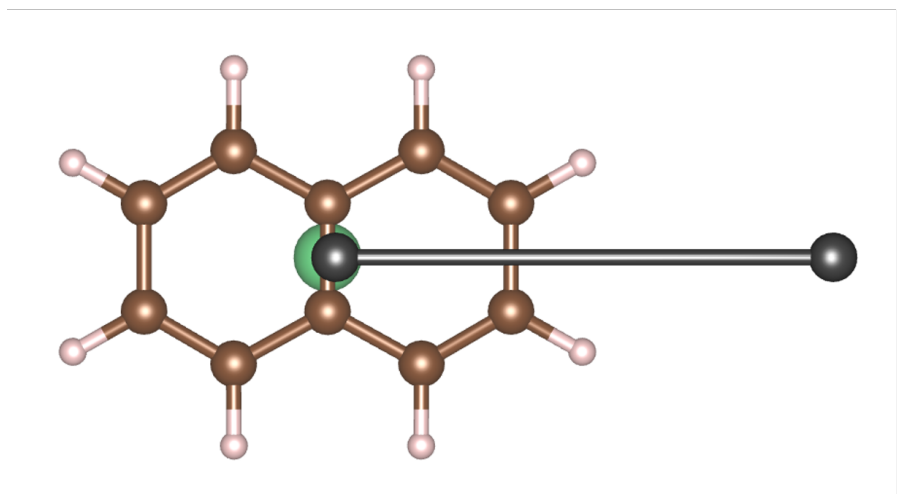

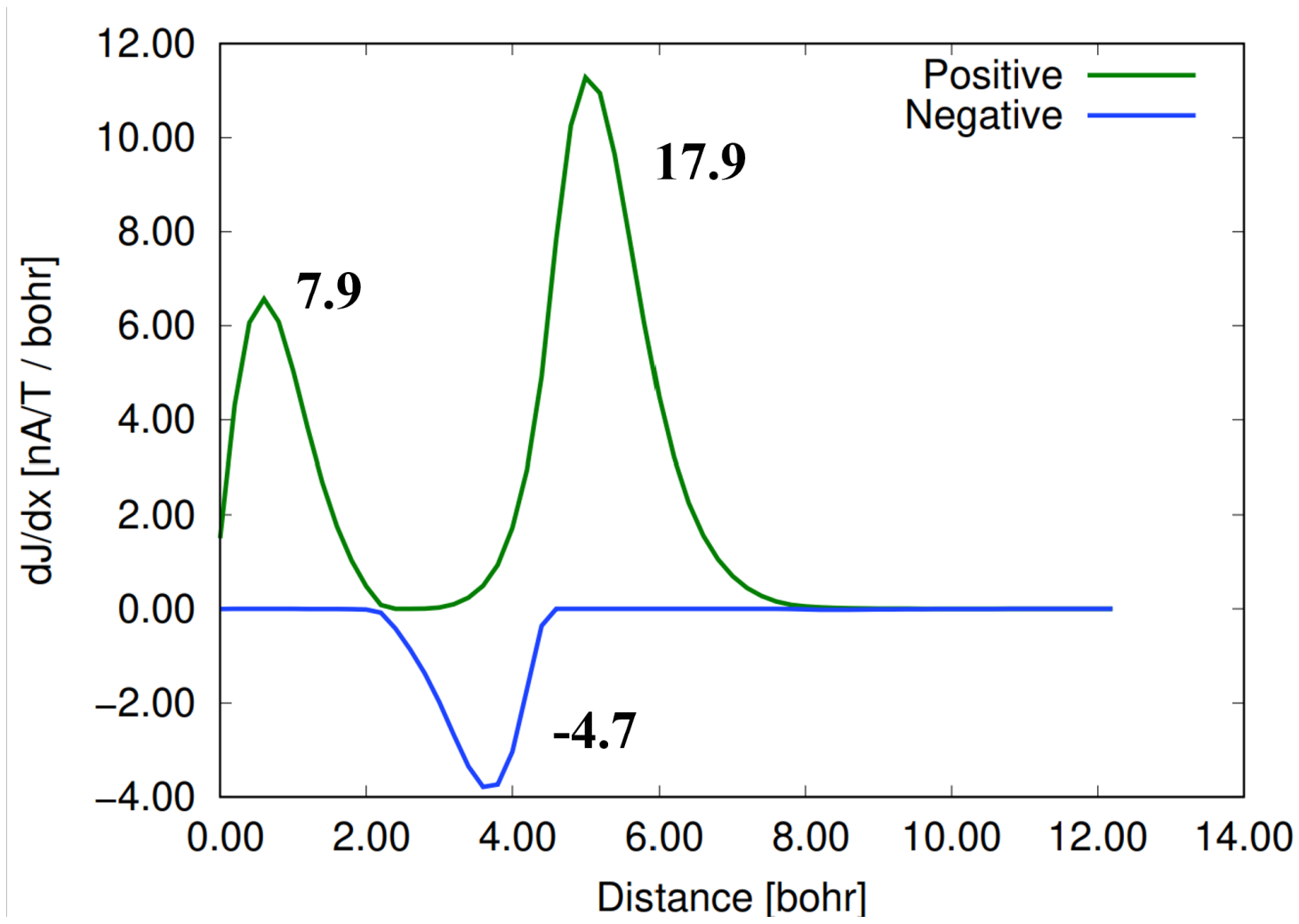

Figure S3: The integration plane, the profile of the magnetically induced ring current (MIRC) of naphthalene and contributions to the MIRC strength in that plane.

Table S1: The molecular orbitals of the naphthalene.

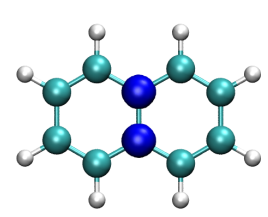	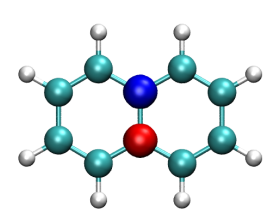	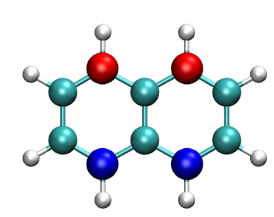
1(a1, core)	2(b2, core)	3(b2, core)
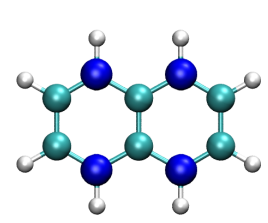	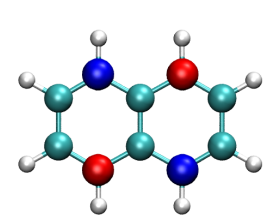	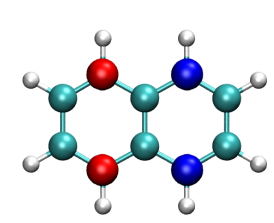
4(a1, core)	5(a2, core)	6(b1, core)

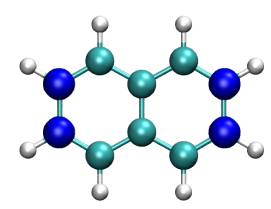

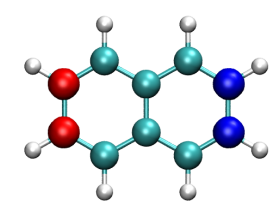

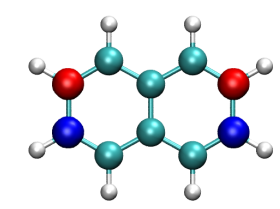

7(a1, core

8(b1, core)

9(b2, core)

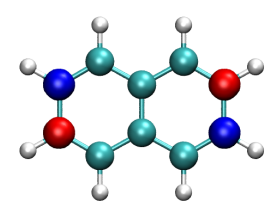

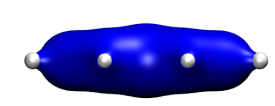

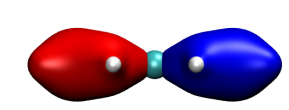

10(a2, core)

11(a1, σ)

12(b1, σ)

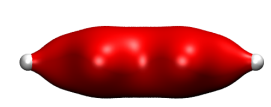

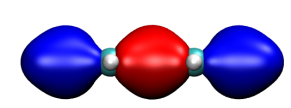

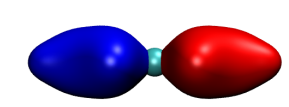

13(b2, σ)

14(a1, σ)

15(a2, σ)

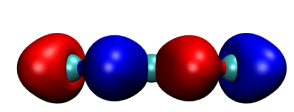

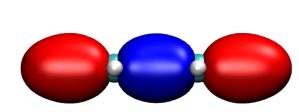

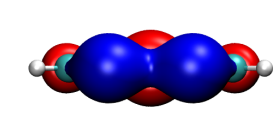

16(b1, σ)

17(b2, σ)

18(a1, σ)

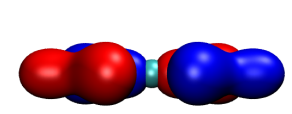

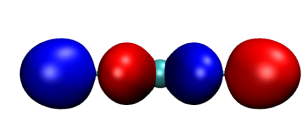

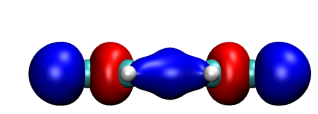

19(b1, σ)

20(a2, σ)

21(a1, σ)

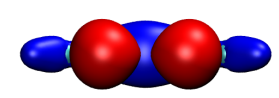

22(b2, σ)

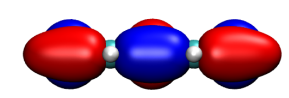

23(a1, σ)

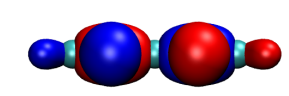

24(a2, σ)

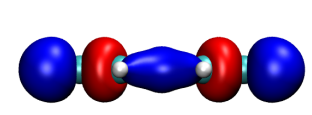

25(b2, σ)

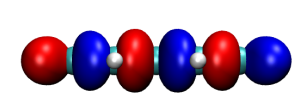

26(b1, σ)

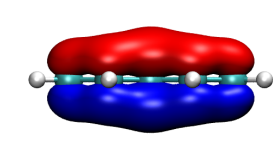

27(a1, π)

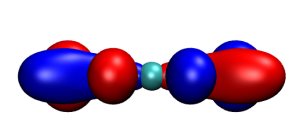

28(b1, σ)

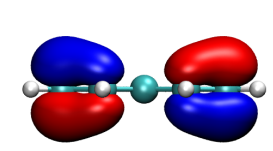

29(b1, π)

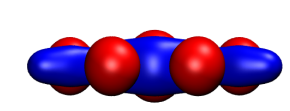

30(a1, σ)

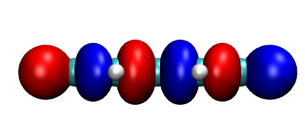

31(a2, σ)

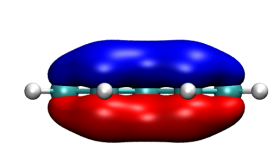

32(b2, π)

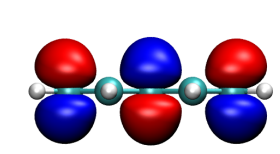

33(a1, π)

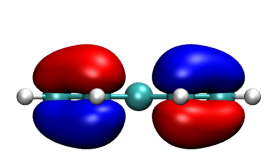

34(a2, π)

1,6-Methanocyclodecapentaene

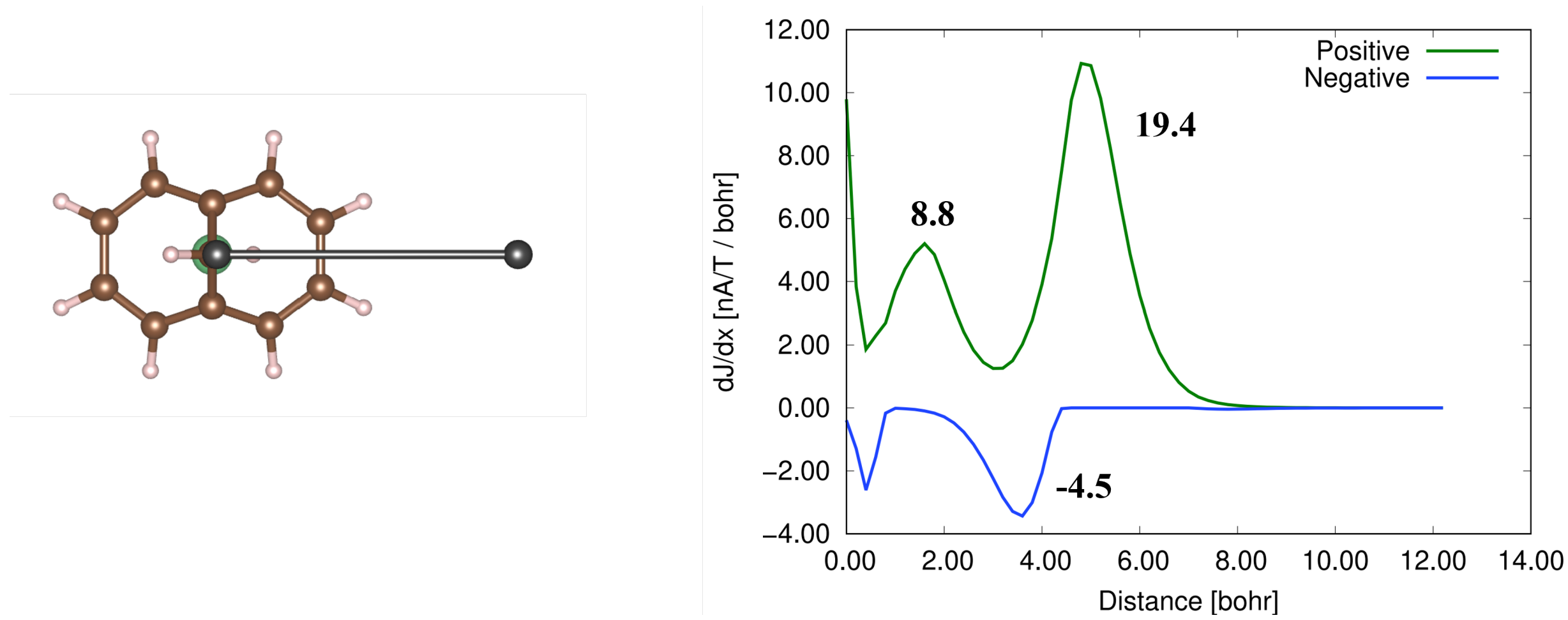

Figure S4: The integration plane, the profile of the magnetically induced ring current (MIRC) of 1,6-methanocyclodecapentaene and contributions to the MIRC strength in that plane.

Table S2: The molecular orbitals of 1,6-methanocyclodecapentaene. The orbitals are assigned as core, σ-like and π-like orbitals.

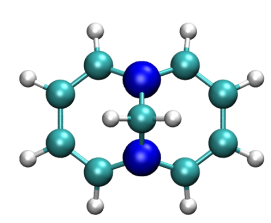

1(a1, core)

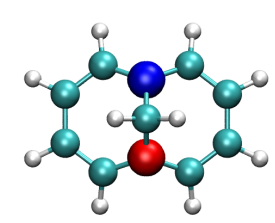

2(b2, core)

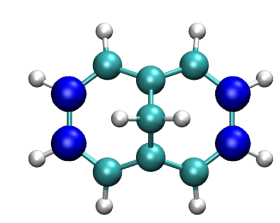

3(a1, core)

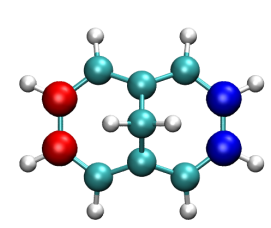

4(b1, core)

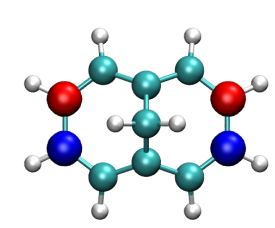

5(b2, core)

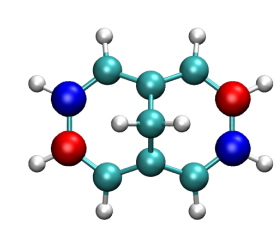

6(a2, core)

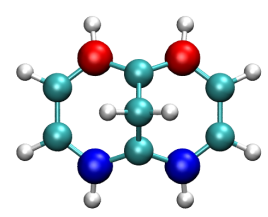
7(b2, core)

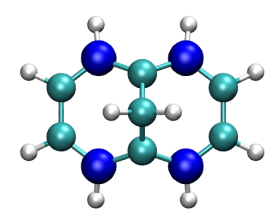
8(a1, core)

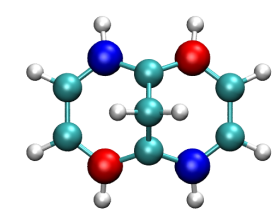
9(a2, core)

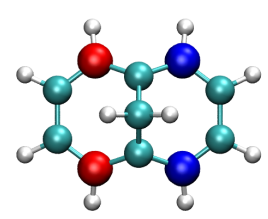
10(b1, core)

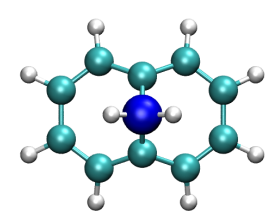
11(a1, core)

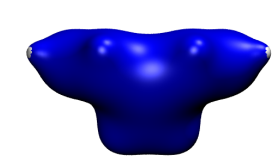
12(a1, σ)

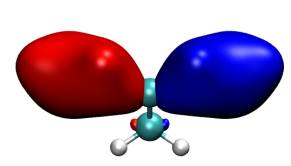
13(b1, σ)

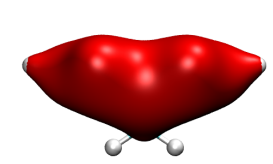
14(b2, σ)

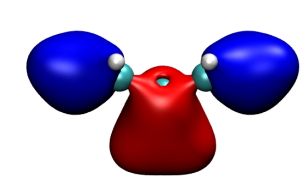
15(a1, σ)

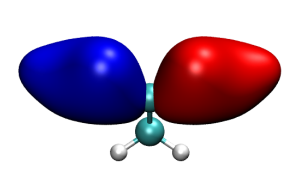
16(a2, σ)

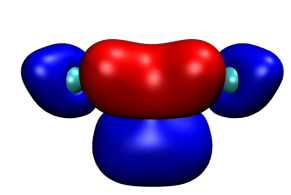
17(a1, σ)

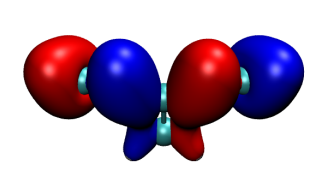
18(b1, σ)

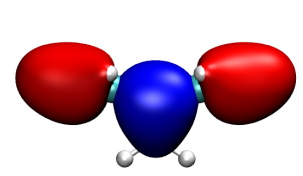
19(b2, σ)

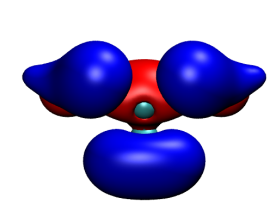
20(a1, σ)

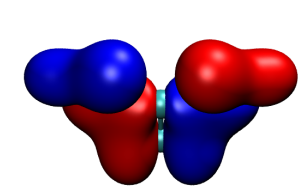
21(b1, σ)

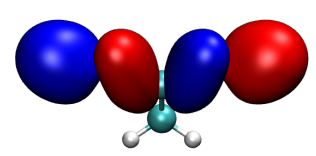
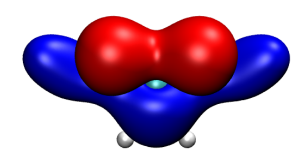
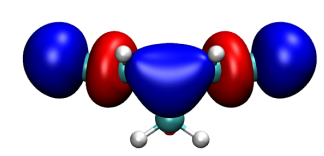

22(a2, σ) 23(b2, σ) 24(a1, σ)

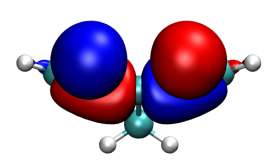
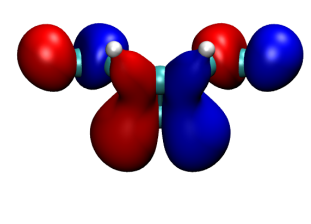
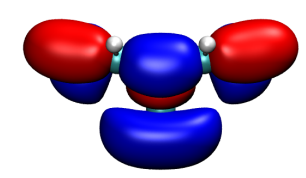

25(a2, σ) 26(b1, σ) 27(a1, σ)

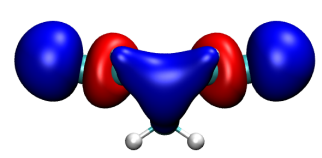
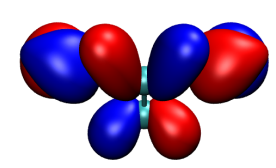
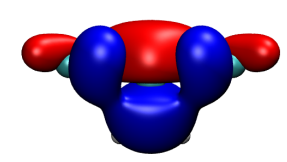

28(b2, σ) 29(b1, σ) 30(b2, σ)

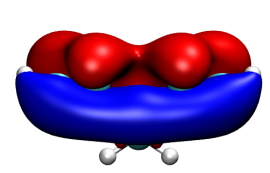
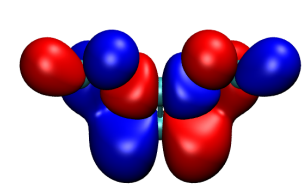
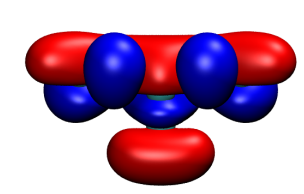

31(a1, π) 32(b1, σ) 33(a1, σ)

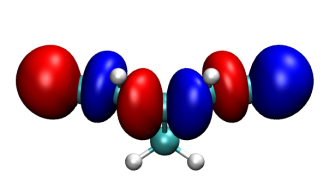
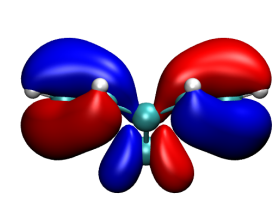
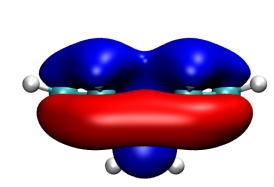

34(a2, σ) 35(b1, π) 36(b2, π)

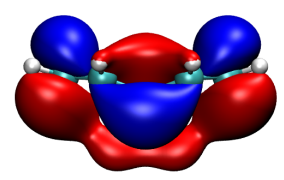

37(a1, σ)

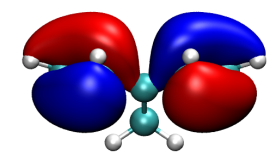

38(a2, π)

$C_9H_9^+$

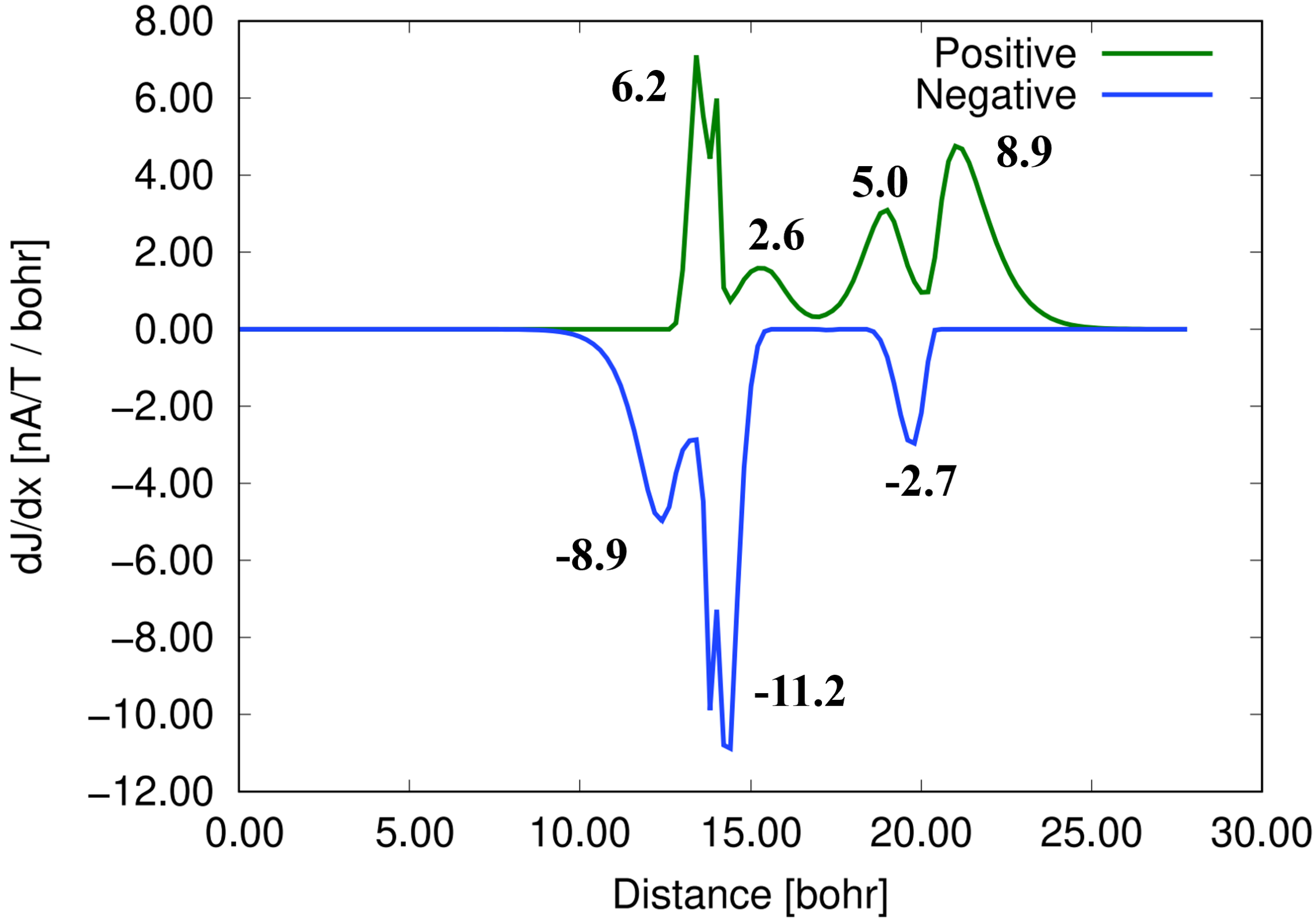

Figure S5: The integrated current-density strength of the singlet state of $C_9H_9{}^+$ calculated at the B3LYP level of theory for each segment of the ring-current profile.

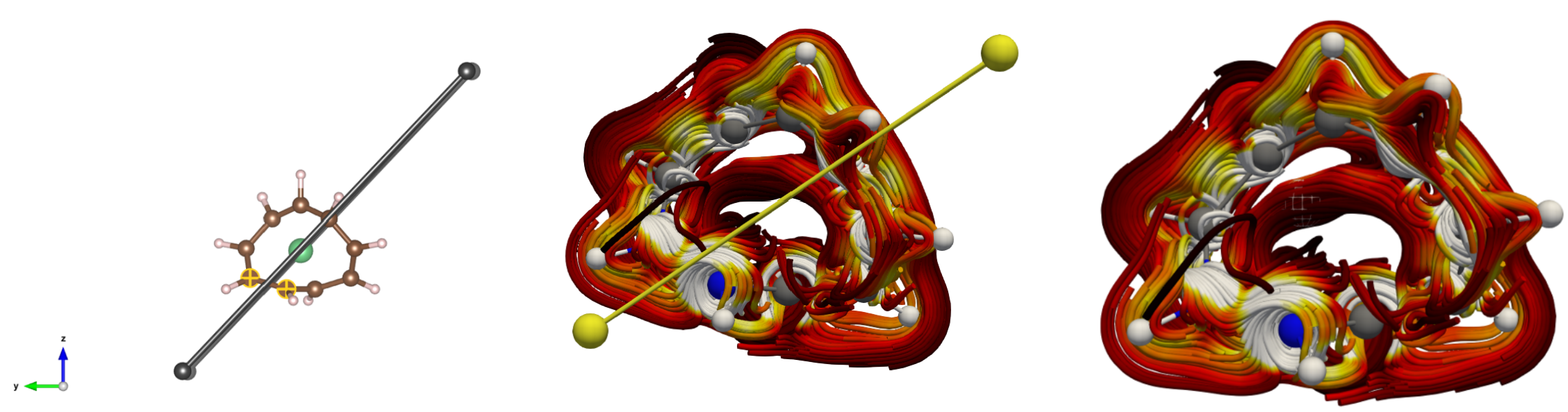

Figure S6: The position of the integration plane (left), the MIRC and the integration plane (middle) and the MIRC (right) of the singlet state of $C_9H_9{}^+$ calculated at the B3LYP level of theory.

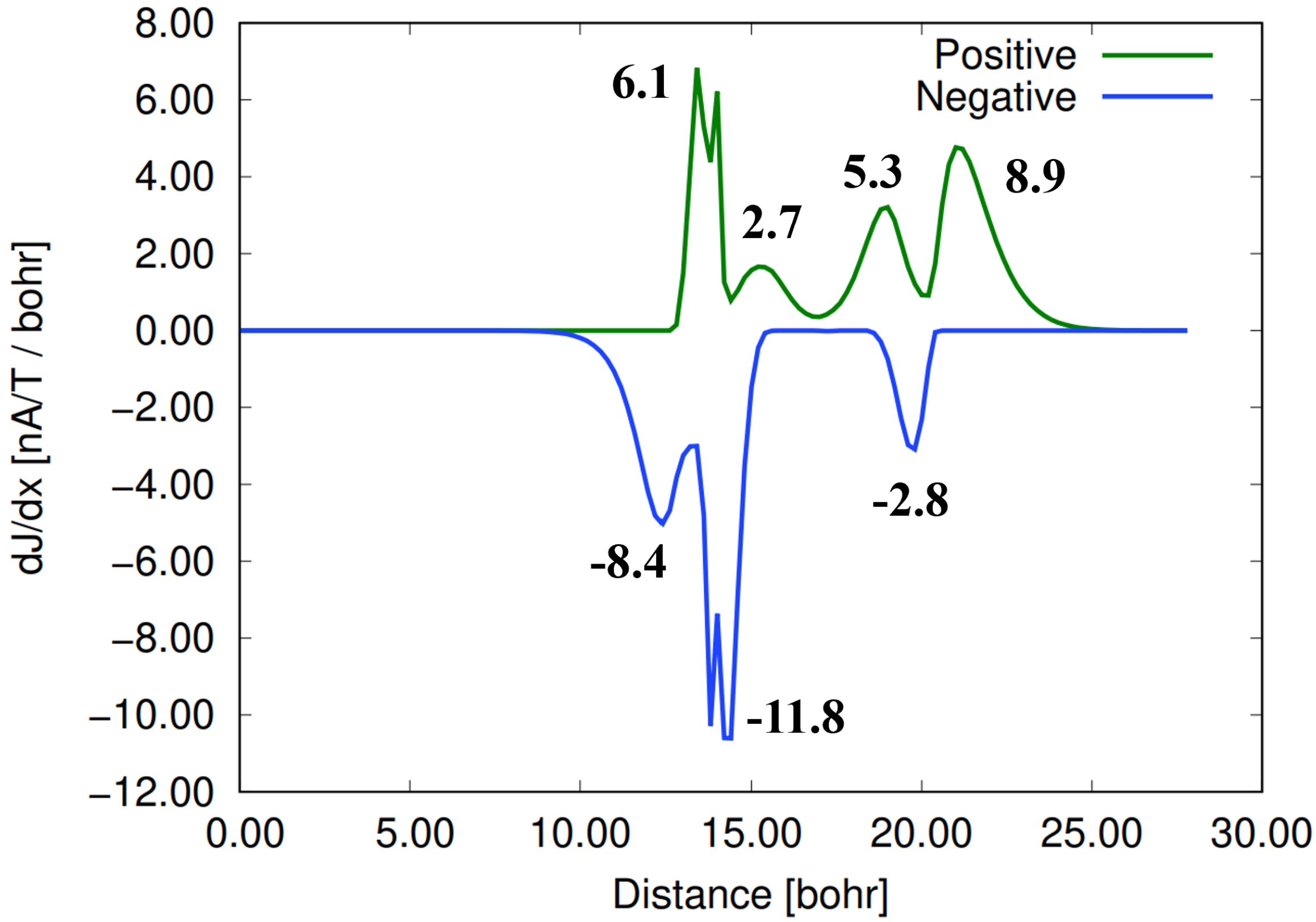

Figure S7: The integrated current-density strength of the singlet state of $C_9H_9^+$ calculated at the ωB97X level of theory for each segment of the ring-current profile.

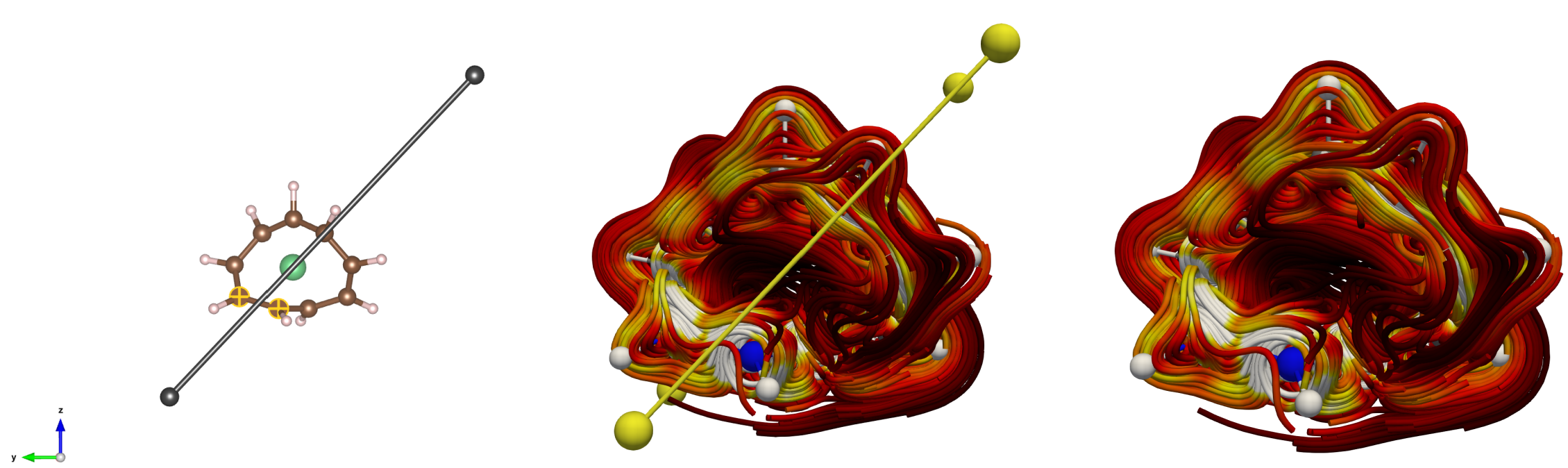

Figure S8: The position of the integration plane (left), the MIRC and the integration plane (middle) and the MIRC (right) of the singlet state of $C_9H_9^+$ calculated at the ωB97X level of theory.

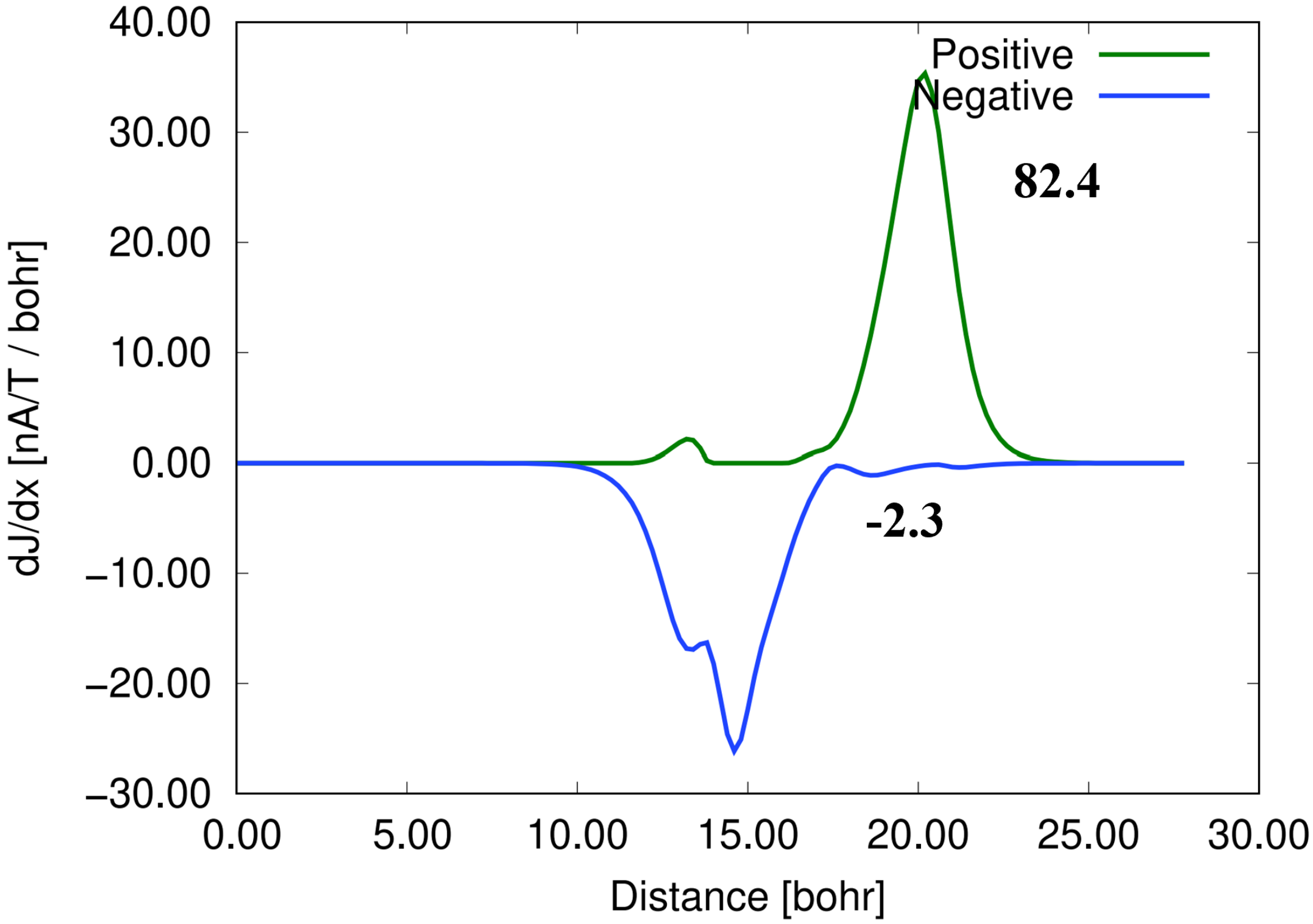

Figure S9: The integrated current-density strength of the triplet state of $C_9H_9{}^+$ calculated at the B3LYP level of theory for each segment of the ring-current profile.

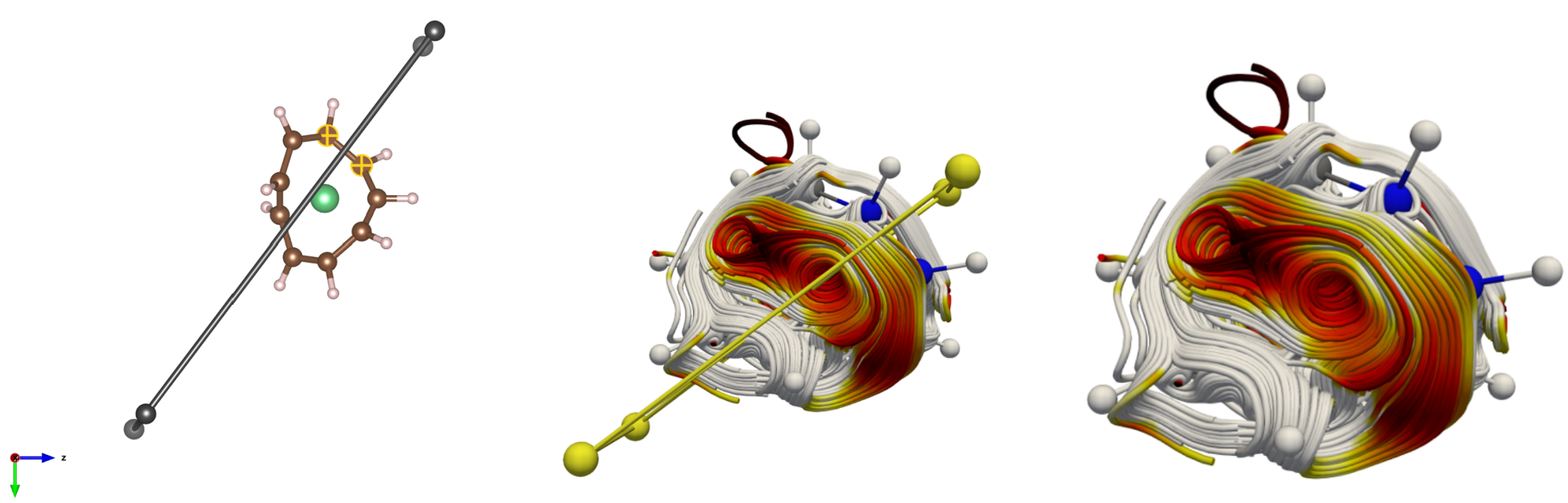

Figure S10: The position of the integration plane (left), the MIRC and the integration plane (middle) and the MIRC (right) of the triplet state of $C_9H_9{}^+$ calculated at the ωB97X level of theory.

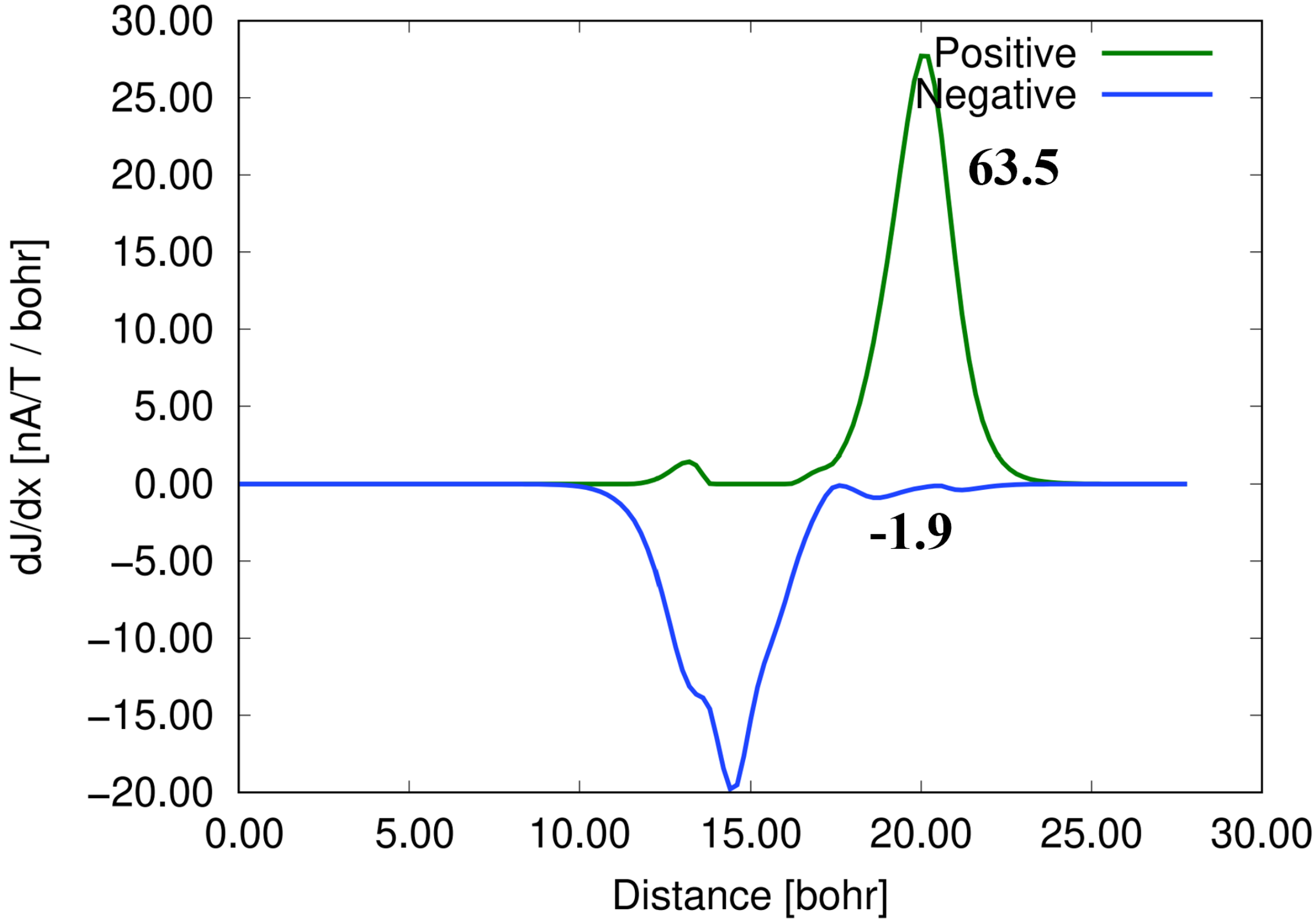

Figure S11: The integrated current-density strength of the triplet state of $C_9H_9{}^+$ calculated at the ωB97X level of theory for each segment of the ring-current profile.

ScC_5H_5

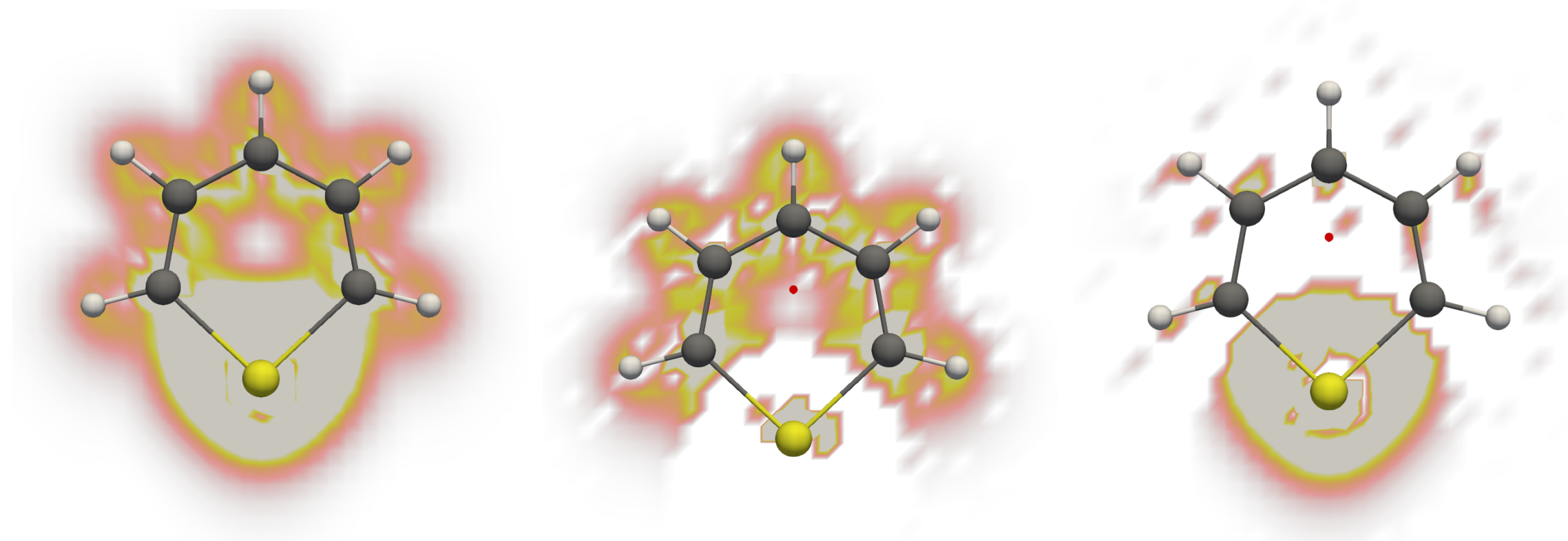

Figure S12: The magnetically induced current density (left), the diatropic contribution (middle) and the paratropic contribution (right) pattern of the singlet ground state of ScC_5H_5.

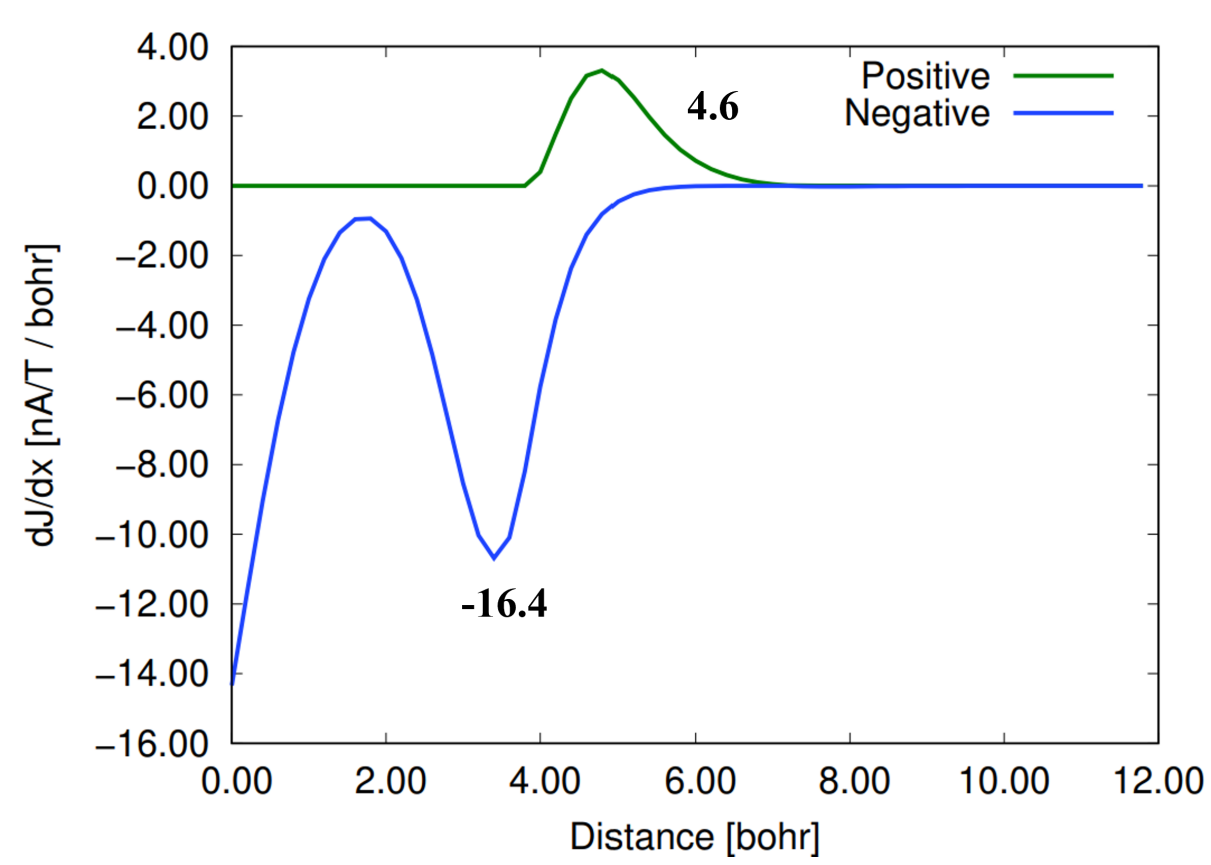

Figure S13: The strength of the MIRC of the singlet ground state of ScC_5H_5.

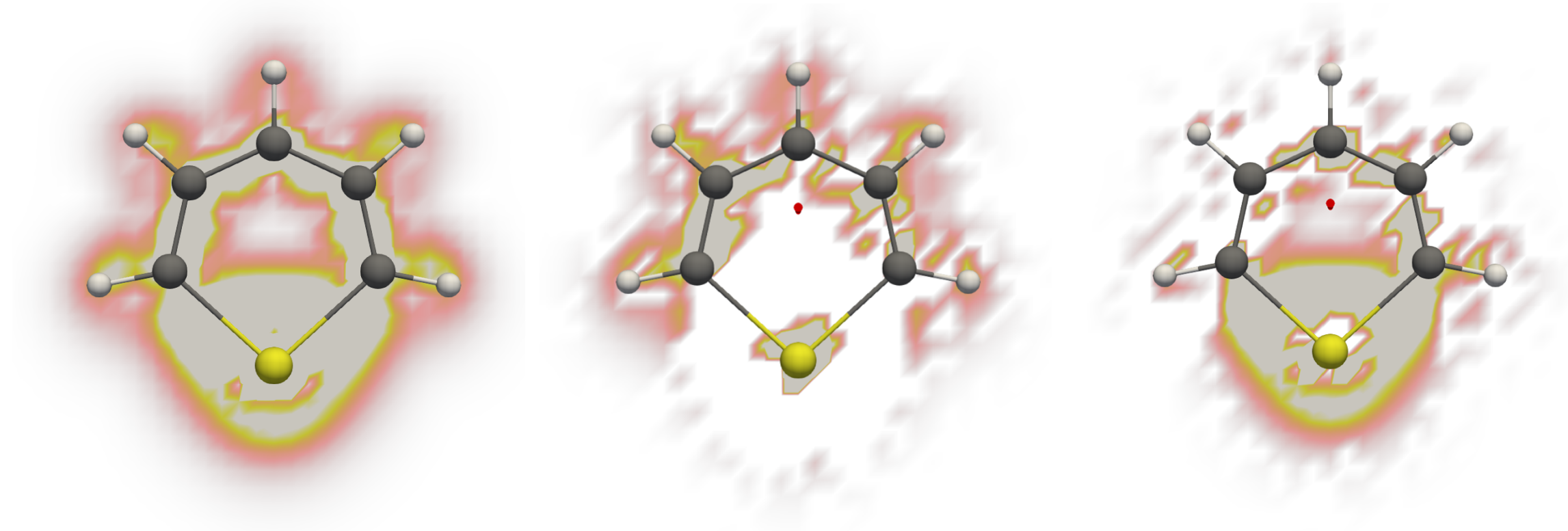

Figure S14: The MIRC (left), the diatropic contribution (middle) and paratropic contribution (right) of the lowest triplet state of ScC_5H_5.

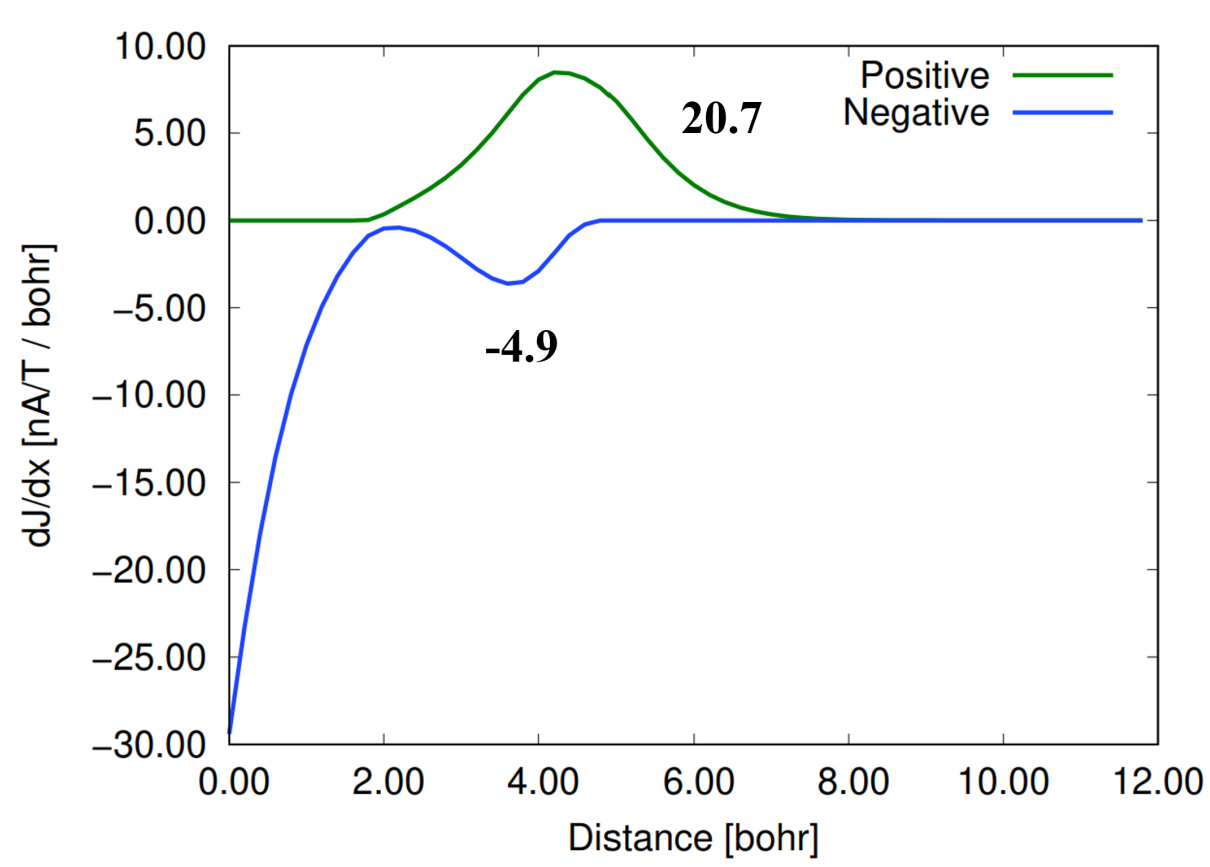

Figure S15: The strength of the MIRC of the T_1 state of ScC_5H_5.

Table S3: Frontier orbitals of the singlet ground state of ScC_5H_5.

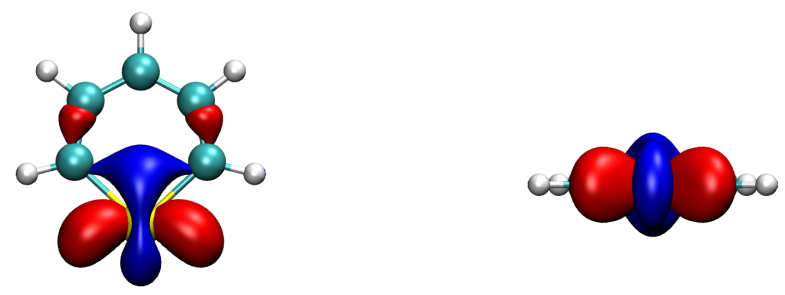	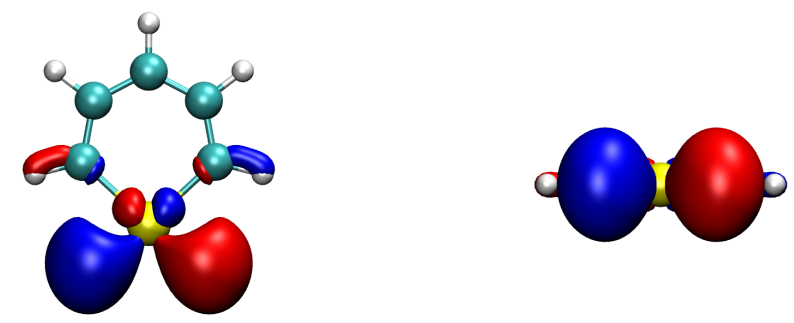
LUMO + 5	LUMO + 4
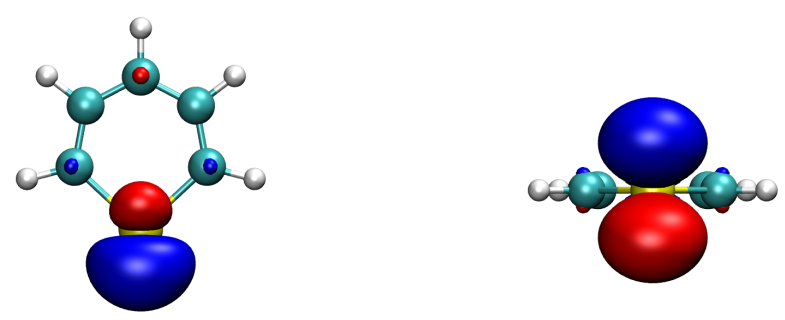	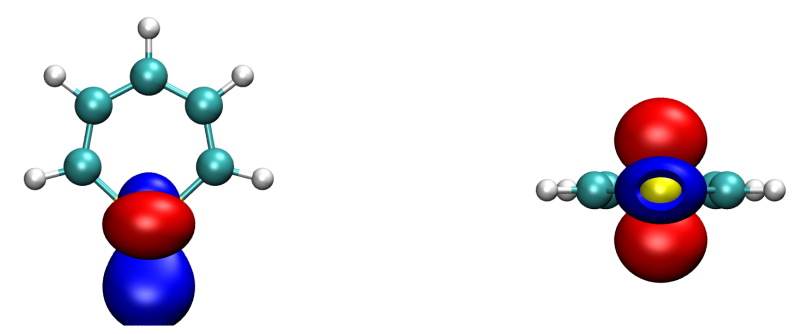
LUMO + 3	LUMO + 2
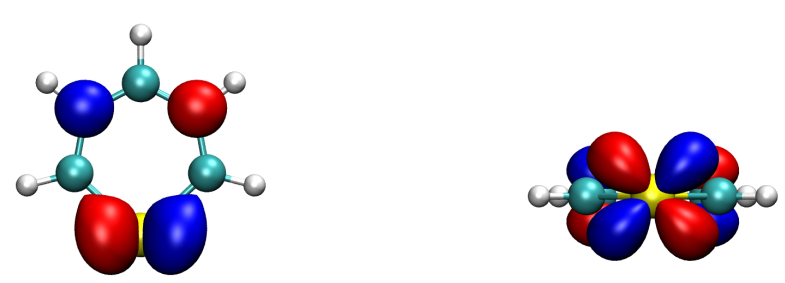	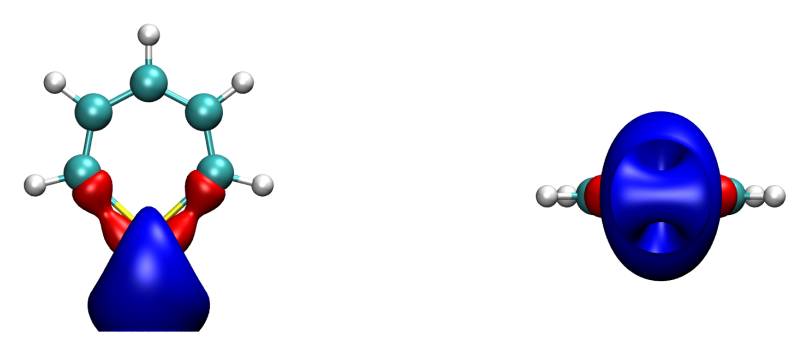
LUMO + 1	LUMO
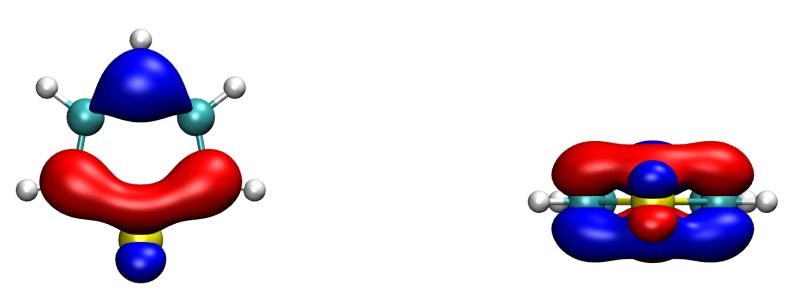	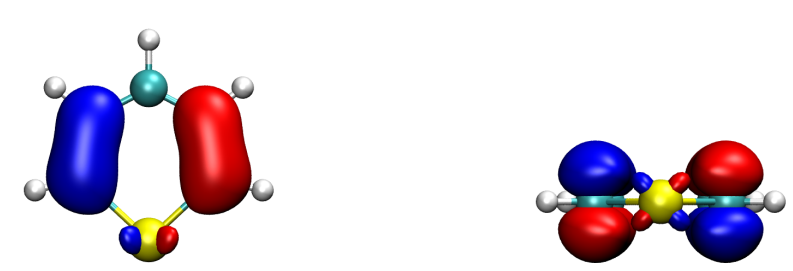
HOMO	HOMO − 1
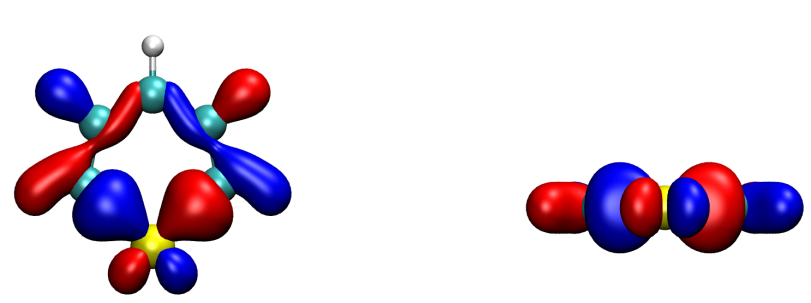	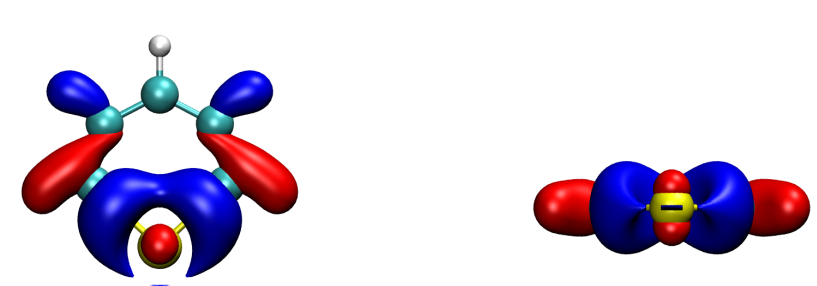
HOMO − 2	HOMO − 3

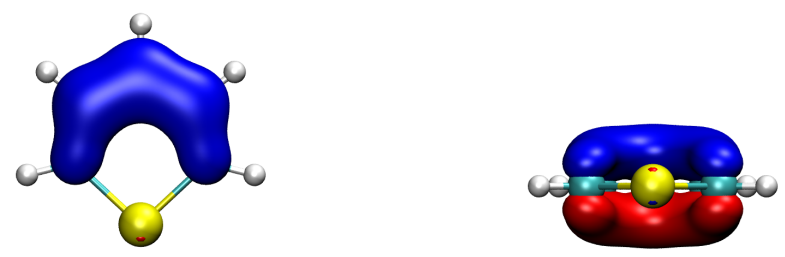

HOMO − 4

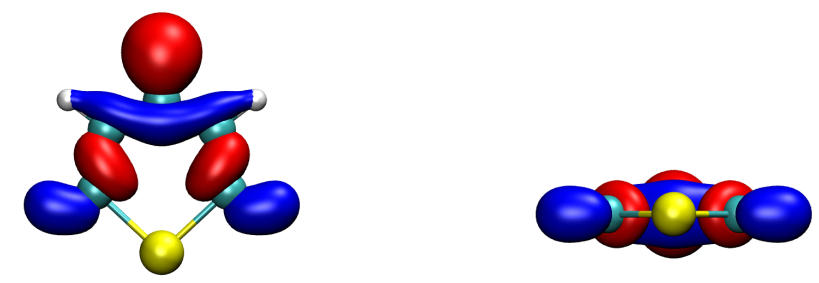

HOMO − 5

Ring-size dependence

Table S4: The total energy (in a.u.), the total energy (in a.u.) per CH unit, the HOMO-LUMO gap (H-L in eV), the C–C distances (in pm) of the C_mH_m rings with m=4n+2 calculated at the B3LYP/def2-TZVP/D4 level.

# C atoms	Energy	Energy/CH	H-L	C–C	C–C
10	-386.772679637	-38.6772680	4.13060	140.53	140.54
14	-541.614267966	-38.6867334	3.54692	139.66	139.67
18	-696.427210117	-38.6904006	2.89502	139.35	139.37
22	-851.228944801	-38.6922248	2.43871	139.20	139.22
26	-1006.024892575	-38.6932651	2.10645	139.11	139.15
30	-1160.817434449	-38.6939145	1.88852	138.14	140.06
34	-1315.607713851	-38.6943590	1.79737	137.28	140.96
38	-1470.397823307	-38.6946796	1.72246	136.91	141.34
42	-1625.186558437	-38.6949181	1.66080	136.71	141.55
46	-1779.974605510	-38.6951001	1.60843	136.59	141.67
50	-1934.762105095	-38.6952421	1.56474	136.50	141.75
54	-2089.549164092	-38.6953549	1.52742	136.45	141.80
58	-2244.335862659	-38.6954459	1.49550	136.41	141.83
62	-2399.122263184	-38.6955204	1.46782	136.38	141.85
66	-2553.908414541	-38.6955820	1.44374	136.36	141.87
70	-2708.694356810	-38.6956337	1.42281	136.34	141.88
74	-2863.480119696	-38.6956773	1.40441	136.33	141.89
78	-3018.265730061	-38.6957145	1.38822	136.32	141.89
82	-3173.051209033	-38.6957465	1.37394	136.32	141.89
86	-3327.836573186	-38.6957741	1.36116	136.31	141.90
90	-3482.621837078	-38.6957982	1.34984	136.31	141.90
94	-3637.407012641	-38.6958193	1.33973	136.30	141.90
98	-3792.192111287	-38.6958379	1.33076	136.30	141.90
110	-4256.547024620	-38.6958820	1.30875	136.29	141.90

Table S5: The strengths of magnetically ring current (in nA/T) of the C_mH_m rings with m=4n+2 calculated using the B3LYP, CAM-B3LYP and ωB97X functionals, the def2-TZVP basis sets and the D4 dispersion term.

# C atoms	B3LYP	CAM-B3LYP	ωB97X
10	14.909916860703193	15.472257257596700	15.747161724443911
14	23.251854296258234	23.715354586249454	24.009256762297877
18	30.633180486252833	31.136328141768555	31.442195812607714
22	37.834529891905099	38.404924284793005	38.733762260664101
26	44.973270819708794	45.615104260269320	45.967410772933484
30	48.121518611724980	33.133934340900410	16.893239530642376
34	41.139742788892846	17.164497978194497	7.1689584696590218
38	35.011981787675310	10.484654748624946	3.7131198147745867
42	29.555092237262954	6.6593224836879532	2.0033421234132942
46	24.876394027124782	4.3168064872736363	1.0975302794313866
50	20.778453757613043	2.8035900468457831	0.61234776441278826
54	17.279762591373789	1.8170452942006918	0.33265057863505876
58	14.279936970310761	1.1758688259356067	0.17641635577676124
62	11.773113257910959	0.75717743989502373	6.717981517689E-002
66	9.6644622623541601	0.48899040939693356	1.836676515588E-002
70	7.9060056881763048	0.31713968404272058	-9.095515815379E-003
74	6.4405394395710189	0.19241844355898363	-2.391735968374E-002
78	5.2254988516436969	0.11666092300463535	-3.162889214241E-002
82	4.2266269320707419	6.832340624211E-002	-3.539963932195E-002
86	3.4027907344363748	3.719922989285E-002	-3.711422465200E-002
90	2.7364170091222944	1.765715708675E-002	-3.755110239654E-002
94	2.1971474614918964	5.556310877747E-003	-3.727608030035E-002
98	1.7500874895463316	-1.657105433370E-003	-3.644605688513E-002

Table S6: The total energy (in au), the total energy (in a.u.) per CH unit, the HOMO-LUMO gap (H-L in eV), the C–C distances (in pm) of the C_mH_m rings with m=4n calculated at the B3LYP/def2-TZVP/D4 level.

# C atoms	Energy	Energy/CH	H-L	C–C	C–C
12	-464.16193118	-38.68016093	2.02007	135.61	146.02
16	-618.99666638	-38.68729165	1.79642	135.67	144.40
20	-773.81050239	-38.69052512	1.64309	135.80	143.57
24	-928.61426172	-38.69226091	1.53532	135.91	143.07
28	-1083.4122856	-38.69329591	1.45671	136.01	142.75
32	-1238.2067365	-38.69396051	1.39878	136.08	142.52
36	-1392.9988202	-38.69441167	1.35568	136.14	142.37
40	-1547.7892592	-38.69473148	1.32251	136.18	142.25
44	-1702.5785119	-38.69496618	1.29726	136.22	142.16
48	-1857.3668826	-38.69514339	1.27824	136.24	142.10
52	-2012.1545813	-38.69528041	1.26389	136.26	142.05
56	-2166.9417569	-38.69538852	1.25297	136.27	142.02
60	-2321.7285184	-38.69547531	1.24438	136.28	141.99
64	-2476.5149464	-38.69554604	1.23819	136.29	141.97
68	-2631.3011026	-38.69560445	1.23314	136.29	141.95
72	-2786.0870342	-38.69565325	1.22934	136.29	141.94
76	-2940.8727783	-38.69569445	1.22661	136.29	141.93
80	-3095.6583642	-38.69572955	1.22447	136.30	141.92
84	-3250.4438155	-38.69575971	1.22279	136.30	141.92
88	-3405.2291512	-38.69578581	1.22167	136.30	141.91
92	-3560.0143871	-38.69580856	1.22068	136.29	141.91
96	-3714.7995359	-38.69582850	1.21983	136.29	141.91
100	-3869.5846085	-38.69584608	1.21929	136.29	141.90

Table S7: The strengths of magnetically ring current (in nA/T) of the C_mH_m rings with m=4n calculated using the B3LYP, CAM-B3LYP and ωB97X functionals, the def2-TZVP basis sets and the D4 dispersion term.

# C atoms	B3LYP	CAM-B3LYP	ωB97X
12	-39.959802005497089	-29.258547525858948	-24.009638226352564
16	-42.786061416922237	-25.817234468558453	-18.520047028836345
20	-42.449276026735802	-21.259974864171987	-13.436285854491839
24	-40.383689108557235	-16.850030032948933	-9.3906190146426347
28	-37.388104568016878	-13.005071319312334	-6.3776694978228479
32	-33.942370868647508	-9.8223924327011822	-4.2120330522881000
36	-30.271328855114501	-7.2719837808490650	-2.7584416056237799
40	-26.729666432644500	-5.2980253495845178	-1.7685903348451435
44	-23.301358010267201	-3.8040633281734908	-1.0754044487976731
48	-20.086521188647918	-2.6911312111437415	-0.65580239360587467
52	-17.142802567618066	-1.8894384038933327	-0.39154313177385119
56	-14.507303817205228	-1.3096230352699449	-0.28652978728624934
60	-12.196070128086948	-0.90278761332107393	-0.18879287350348295
64	-10.168036778644201	-0.62290780982408023	-0.12226458569310612
68	-8.4382513387162188	-0.42841150571073644	-9.416955530364E-002
72	-6.9623655204076158	-0.29564231448295908	-6.451466273543E-002
76	-5.7119190014608439	-0.20560008277515288	-5.081630858764E-002
80	-4.6688074166527747	-0.13895591630375240	-4.233501399720E-002
84	-3.8014169637285433	-9.795286250776E-002	-4.617136450171E-002
88	-3.0830393825759232	-7.047415448547E-002	-4.274260319186E-002
92	-2.4943540735849798	-5.170911138520E-002	-4.040654401734E-002
96	-2.0136424337982826	-3.890981568412E-002	-3.865548959203E-002
100	-1.6220967463578495	-2.998591660781E-002	-3.409094649463E-002

Table S8: The strengths of magnetically ring current (I in nA/T) of the aromatic (4n+2) C_mH_m and antiaromatic (4n) rings calculated at the HF/def2-TZVP and MP2/def2-TZVP levels.

Aromatic			Antiaromatic		
Molecule	I(MP2)	I(HF)	Molecule	I(MP2)	I(HF)
$C_{10}H_{10}$	15.38	17.49	$C_{12}H_{12}$	-32.24	-19.11
$C_{14}H_{14}$	23.60	26.29	$C_{16}H_{16}$	-28.19	-13.74
$C_{18}H_{18}$	31.70	34.34	$C_{20}H_{20}$	-22.01	-9.29
$C_{22}H_{22}$	38.89	42.30	$C_{24}H_{24}$	-16.82	-6.03
$C_{26}H_{26}$	83.10	24.73	$C_{28}H_{28}$	-12.45	-3.80
$C_{30}H_{30}$	35.24	10.31			

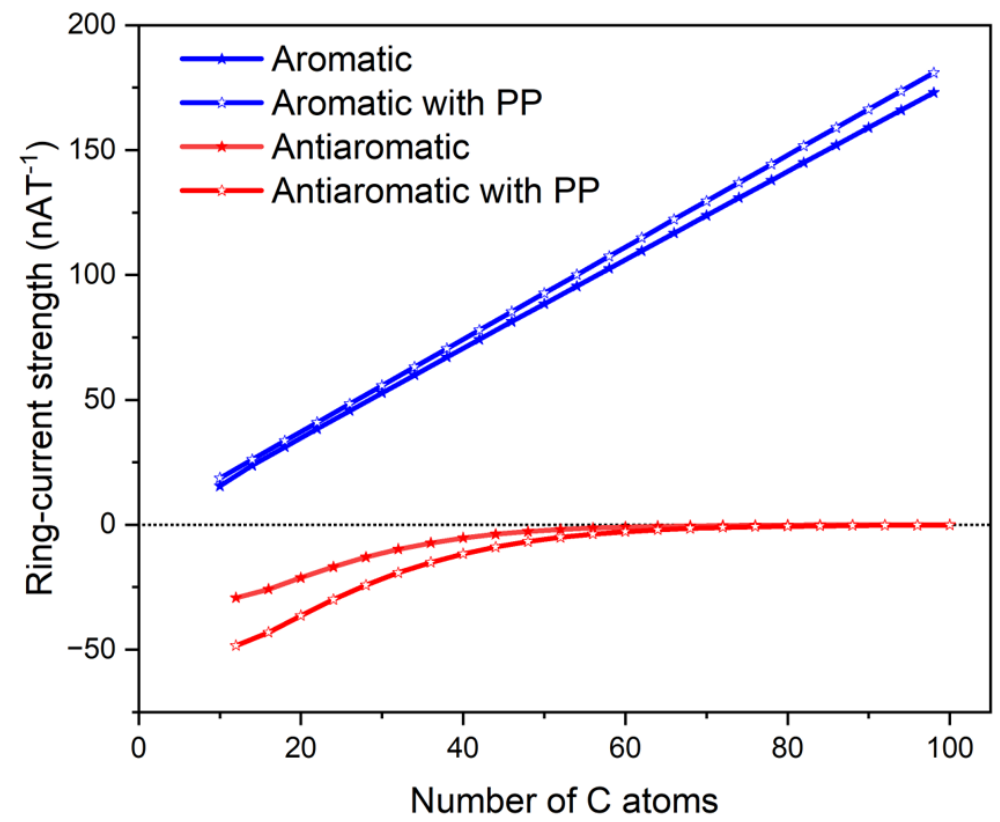

Figure S16: The MIRC strength (in nA/T) of aromatic *trans*-annulene rings with equidistant C–C distances as a function of the number of carbon atoms. The MIRC strengths (in nA/T) of the antiaromatic *trans*-annulene rings have been calculated for optimized molecular rings with alternating C–C distances. The MIRC strengths have been calculated at the all-electron B3LYP level as well at the pseudo-π level using the B3LYP functional.

Cartesian coordinates of the Ni-norcorrole trimer

The molecular structure is optimized at the B3LYP/def2-TZVP/D4 level.

```
99

Ni     0.0000000    0.0000000    0.0000000
N      1.7681799   -0.2744407    0.0000000
C      2.5625484    0.8060480    0.0000000
C     -2.4536443    1.4353422    0.0000000
C      3.9116092    0.3198918    0.0000000
C     -3.8498119    1.0478264    0.0000000
H      4.7996833    0.9280779    0.0000000
H     -4.6922291    1.7212443    0.0000000
N      0.4524272    1.7313707    0.0000000
N     -1.7682773    0.2661201    0.0000000
N     -0.4505968   -1.7359922    0.0000000
C      0.3514637   -2.8239927    0.0000000
C     -2.5619719   -0.8155121    0.0000000
C     -0.3505051    2.8185498    0.0000000
C      1.7622854    2.0371460    0.0000000
C      2.4543329   -1.4446319    0.0000000
C     -1.7600955   -2.0441049    0.0000000
C     -0.5337660   -3.9519121    0.0000000
C     -3.9118142   -0.3303741    0.0000000
```

C	0.5356464	3.9456216	0.0000000
C	1.8410396	3.4621780	0.0000000
C	3.8519189	-1.0584826	0.0000000
C	-1.8386960	-3.4692419	0.0000000
H	-0.2360947	-4.9879153	0.0000000
H	-4.8036878	-0.9361418	0.0000000
H	0.2388315	4.9818135	0.0000000
H	2.7399177	4.0569541	0.0000000
H	4.6947874	-1.7318800	0.0000000
H	-2.7379166	-4.0637106	0.0000000
C	1.7679791	-2.6725860	0.0000000
C	-1.7675264	2.6639856	0.0000000
H	2.3611474	-3.5774278	0.0000000
H	-2.3625944	3.5674511	0.0000000
Ni	0.0000000	0.0000000	2.8893076
N	1.7902760	0.1045401	3.0420056
C	2.4568317	-1.0416452	3.2130733
C	2.5859233	1.1984988	3.2061229
C	3.8225293	-0.7009549	3.4727534
C	3.9057517	0.6797811	3.4614037
H	4.6307645	-1.3918099	3.6480869
H	4.7972351	1.2639203	3.6290086
N	0.2685246	-1.7715153	3.0482259
N	-1.7892480	-0.0987168	3.0518066
N	-0.2649329	1.7744535	3.0469953
C	-0.6348802	-2.7711311	3.2169039
C	-2.4527847	1.0495432	3.2230294
C	0.6388169	2.7758413	3.2035163
C	1.5318146	-2.1910605	3.2056109
C	-2.5837387	-1.1910627	3.2280362
C	-1.5269380	2.1972801	3.2053491
C	0.1365872	-3.9570620	3.4445389
C	-3.8168929	0.7129903	3.4947302
C	-0.1298065	3.9639431	3.4249611
C	1.4802534	-3.5954279	3.4430371
C	-3.9007507	-0.6681667	3.4914078
C	-1.4734784	3.6036928	3.4313474
H	-0.2577227	-4.9482538	3.6030071
H	-4.6228377	1.4059844	3.6735736
H	0.2676782	4.9554296	3.5735673
H	2.3195059	-4.2506813	3.6111997
H	-4.7903899	-1.2504158	3.6695223
H	-2.3108199	4.2621026	3.5959561
C	-2.0204115	-2.4794712	3.2744595
C	2.0243833	2.4864579	3.2515178

H	-2.6932103	-3.3107929	3.4463177
H	2.6992638	3.3180612	3.4128646
Ni	0.0000000	0.0000000	-2.8893076
N	1.7902760	0.1045401	-3.0420056
C	2.4568317	-1.0416452	-3.2130733
C	2.5859233	1.1984988	-3.2061229
C	3.8225293	-0.7009549	-3.4727534
C	3.9057517	0.6797811	-3.4614037
H	4.6307645	-1.3918099	-3.6480869
H	4.7972351	1.2639203	-3.6290086
N	0.2685246	-1.7715153	-3.0482259
N	-1.7892480	-0.0987168	-3.0518066
N	-0.2649329	1.7744535	-3.0469953
C	-0.6348802	-2.7711311	-3.2169039
C	-2.4527847	1.0495432	-3.2230294
C	0.6388169	2.7758413	-3.2035163
C	1.5318146	-2.1910605	-3.2056109
C	-2.5837387	-1.1910627	-3.2280362
C	-1.5269380	2.1972801	-3.2053491
C	0.1365872	-3.9570620	-3.4445389
C	-3.8168929	0.7129903	-3.4947302
C	-0.1298065	3.9639431	-3.4249611
C	1.4802534	-3.5954279	-3.4430371
C	-3.9007507	-0.6681667	-3.4914078
C	-1.4734784	3.6036928	-3.4313474
H	-0.2577227	-4.9482538	-3.6030071
H	-4.6228377	1.4059844	-3.6735736
H	0.2676782	4.9554296	-3.5735673
H	2.3195059	-4.2506813	-3.6111997
H	-4.7903899	-1.2504158	-3.6695223
H	-2.3108199	4.2621026	-3.5959561
C	-2.0204115	-2.4794712	-3.2744595
C	2.0243833	2.4864579	-3.2515178
H	-2.6932103	-3.3107929	-3.4463177
H	2.6992638	3.3180612	-3.4128646
